# Confinement physical entanglement enables programmable polymeric mesoporous membranes from nanocrystal superlattices

**DOI:** 10.1093/nsr/nwag323

**Published:** 2026-05-30

**Authors:** Zhebin Zhang, Ting Wang, Yutong Gao, Yifan Gao, Dong Yang, Tongtao Li, Wei Li, Angang Dong

**Affiliations:** State Key Laboratory of Molecule Engineering of Polymers and Department of Macromolecular Science, Fudan University, Shanghai 200438, China; State Key Laboratory of Porous Materials for Separation and Conversion, Department of Chemistry, and Shanghai Key Laboratory of Molecular Catalysis and Innovative Materials, Fudan University, Shanghai 200438, China; State Key Laboratory of Porous Materials for Separation and Conversion, Department of Chemistry, and Shanghai Key Laboratory of Molecular Catalysis and Innovative Materials, Fudan University, Shanghai 200438, China; State Key Laboratory of Molecule Engineering of Polymers and Department of Macromolecular Science, Fudan University, Shanghai 200438, China; State Key Laboratory of Molecule Engineering of Polymers and Department of Macromolecular Science, Fudan University, Shanghai 200438, China; State Key Laboratory of Porous Materials for Separation and Conversion, Department of Chemistry, and Shanghai Key Laboratory of Molecular Catalysis and Innovative Materials, Fudan University, Shanghai 200438, China; Southwest Research & Design Institute of the Chemical Industry, Chengdu 610225, China; State Key Laboratory of Porous Materials for Separation and Conversion, Department of Chemistry, and Shanghai Key Laboratory of Molecular Catalysis and Innovative Materials, Fudan University, Shanghai 200438, China; Southwest Research & Design Institute of the Chemical Industry, Chengdu 610225, China; State Key Laboratory of Porous Materials for Separation and Conversion, Department of Chemistry, and Shanghai Key Laboratory of Molecular Catalysis and Innovative Materials, Fudan University, Shanghai 200438, China; Southwest Research & Design Institute of the Chemical Industry, Chengdu 610225, China

**Keywords:** mesoporous membranes, nanocrystals, polymer entanglement, superlattices

## Abstract

Mesoporous membranes with tunable architectures and robust mechanical properties remain challenging to fabricate, owing to the difficulty of simultaneously achieving structural order and mechanical stability. We report a general strategy to construct programmable polymeric mesoporous membranes by exploiting confined physical entanglement within polymer-grafted nanocrystal (NC) superlattices. Two-dimensional superlattices, self-assembled at the liquid-air interface from size-, shape-, and composition-controlled NCs, serve as structural templates, and thermal annealing activates polymer entanglement to stabilize the superlattice framework. Subsequent selective removal of NC cores yields free-standing, long-range-ordered polymeric mesoporous membranes that exhibit remarkable specific moduli and deformability. Importantly, this approach enables independent control over pore size, wall thickness, and pore symmetry, offering precise structural programmability beyond conventional templating methods. This strategy is compatible with a wide range of building blocks and binary superlattice configurations, enabling the rational design of mechanically robust mesoporous membranes with hierarchical structural order.

## Teaser

Polymeric mesoporous membranes are constructed via confinement-induced physical entanglement of polymer ligands within nanocrystal superlattices, enabling precise structural programmability and enhanced specific modulus.

## INTRODUCTION

Colloidal nanocrystals (NCs), with their highly tunable size, shape, and composition, serve as versatile building blocks for constructing ordered superlattices that exhibit collective properties beyond those of individual NCs [1,2]. Among them, two-dimensional nanocrystal superlattices (2DSLs) assembled at interfaces provide model systems for studying collective phenomena and serve as promising templates for constructing functional membranes and hierarchical materials with nanoscale precision [3,4]. Functionalization of NCs with polymer ligands provides a further level of control and expands the design space of such assemblies by introducing soft-matter characteristics such as structural adaptability and mechanical compliance [5–8]. However, the inherent flexibility of the grafted polymer ligands renders 2DSLs mechanically soft and fragile, limiting their structural stability and making it challenging to preserve mesoscale order during processing [9,10]. In bulk polymer systems, physical entanglement of polymer chains is known to enhance mechanical properties in elastomers, gels, and other polymeric materials [11–14]. Yet, the manifestation and contribution of physical entanglement to the mechanical properties of polymer-grafted NC superlattices, where polymer chains are confined to interparticle gaps and curved nanoscale interfaces, remain largely unexplored.

Mesoporous materials, featuring ordered pores in the 2–50 nm range, have attracted broad interest owing to their high surface areas and tunable architectures [15,16]. While soft-templating strategies based on micellar self-assembly have enabled the construction of diverse mesostructures, mesoporous polymers remain limited in compositional diversity [17,18]. Most reported systems are restricted to phenolic resins or polydopamine, whose intrinsic crosslinking capability facilitates structural retention after template removal [19–21]. In contrast, polystyrene (PS), a representative commercial thermoplastic lacking inherent crosslinking functionality, offers excellent processability, chemical stability, and compatibility with diverse functional modifications [22,23]. However, its absence of intrinsic crosslinking, together with high chain mobility, often results in structural collapse during template removal, making it difficult to preserve ordered porosity, particularly in free-standing or ultrathin membranes [24–26]. Therefore, developing a general strategy to stabilize and structurally program mesoporous PS-based membranes remains an important yet unresolved challenge.

Herein, we report a general approach to fabricate programmable polymeric mesoporous membranes (PMMs) by leveraging confinement-induced physical entanglement within polymer-grafted NC superlattices. Ordered 2DSLs are constructed from polymer-functionalized NCs with tunable size, shape, and composition. Subsequent thermal annealing activates confined entanglement of polymer ligands, stabilizing the superlattice framework. Selective removal of NC cores yields free-standing PMMs that preserve long-range order and exhibit high specific modulus at low density. Notably, this modular NC-based strategy enables independent control over mesopore size, wall thickness, and pore symmetry through the NC diameter, polymer molecular weight, and superlattice symmetry. The strategy accommodates diverse NC building blocks and binary superlattice assemblies, offering a general framework for constructing hierarchically organized mesoporous membranes with enhanced mechanical integrity.

## RESULTS AND DISCUSSION

**Figure 1.**
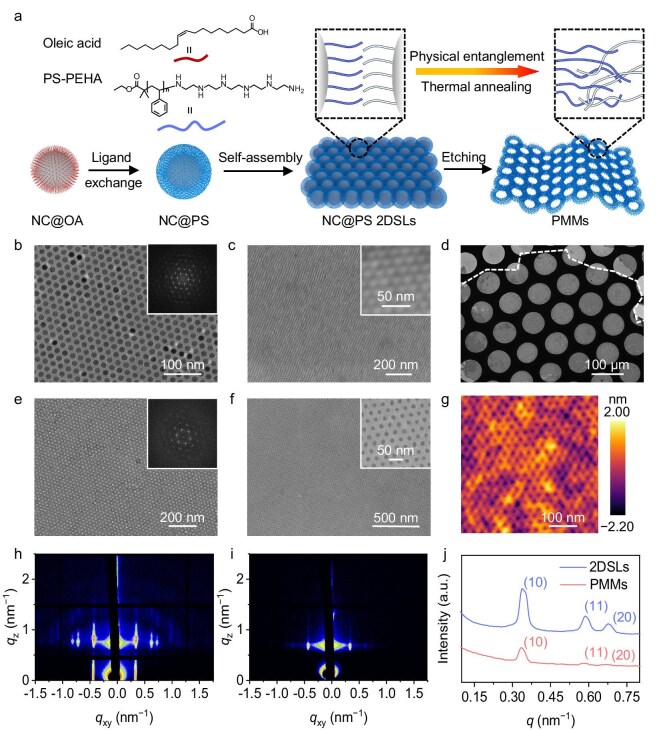
(a) Schematic illustration of the fabrication of two-dimensional nanocrystal superlattices (2DSLs) and programmable polymeric mesoporous membranes (PMMs). (b) Transmission electron microscopy (TEM) and (c) scanning electron microscopy (SEM) images of 20@13k-2DSLs. (d) Low- and (e) high-magnification TEM images of 20@13k-PMMs. (f) SEM and (g) atomic force microscopy (AFM) images of 20@13k-PMMs. (h and i) 2D grazing-incidence small-angle X-ray scattering (GISAXS) patterns of 20@13k-2DSLs and 20@13k-PMMs, respectively, and (j) the corresponding 1D integrated GISAXS curves. Insets in TEM images show corresponding FFT patterns, and insets in SEM images show high-magnification views.

As schematically illustrated in Fig. 1a, PMMs were constructed through a sequential process including NC surface functionalization, interfacial self-assembly, thermal treatment, and selective template removal. Oleic acid-capped Fe_3_O_4_ NCs (NC@OA) were first synthesized (Fig. S1) and subsequently subjected to ligand exchange with pentaethylenehexamine-terminated polystyrene (PS-PEHA) to yield polymer-grafted NCs (NC@PS). These NC@PS building blocks were assembled at the liquid–air interface into ordered 2DSLs, followed by thermal annealing to activate the grafted polymer chains and induce confined physical entanglement within the superlattice framework. Subsequent selective etching of the NC cores produced continuous polymeric membranes with well-defined mesoporous architectures.

As a representative example, PS-PEHA with a number-average molecular weight (*M*_n_) of 13 kDa was grafted onto 20 nm Fe_3_O_4_ NCs, designated as 20@13k. The corresponding 20@13k readily formed highly ordered 2DSLs (20@13k-2DSLs), exhibiting long-range ordered hexagonal packing. Transmission electron microscopy (TEM) reveals uniform interparticle spacing and periodic ordering, while the corresponding fast Fourier transform (FFT) patterns confirm the hexagonal symmetry of the superlattices (Fig. 1b). Consistent ordering over large areas is further evidenced by scanning electron microscopy (SEM) images (Fig. 1c). The formation of such long-range ordered superlattices also indirectly verifies the successful polymer grafting, as homogeneous polymer shells are required to ensure colloidal stability and cooperative assembly.

The as-assembled 20@13k-2DSLs were thermally annealed at 180°C for 30 min to activate confined physical entanglement of the grafted polymer ligands between neighboring NCs, thereby stabilizing the superlattice framework. Subsequent selective etching of the Fe_3_O_4_ cores yielded free-standing PMMs, hereafter referred to as 20@13k-PMMs. Low-magnification TEM image (Fig. 1d) shows that the resulting membranes retain structural integrity over areas exceeding 10 000 μm^2^, with continuous coverage and no visible cracks or defects. High-magnification TEM images (Fig. 1e) reveal uniformly distributed and periodically arranged mesopores, and the FFT patterns confirm that the hexagonal symmetry of the structure is preserved after etching. These observations are further supported by SEM and atomic force microscopy (AFM) images (Fig. 1f, g), which demonstrate homogeneous pore size and long-range ordering across the membrane surface. In addition, AFM analysis reveals a root-mean-square roughness of approximately 0.57 nm, indicating a smooth membrane surface at the nanoscale.

Grazing-incidence small-angle X-ray scattering (GISAXS) measurements were performed to further probe the long-range ordering of the assemblies. The two-dimensional GISAXS pattern of the 20@13k-2DSLs exhibits well-defined diffraction spots, indicative of highly ordered in-plane packing of the NCs (Fig. 1h). After removal of the NC cores, the 20@13k-PMMs exhibit a reduced number and intensity of diffraction spots (Fig. 1i), which can be attributed to the partial loss of scattering contrast upon etching. One-dimensional scattering profiles obtained by azimuthal integration of the GISAXS patterns (Fig. 1j) show three characteristic peaks with position ratios of 1:$\sqrt 3 $:2, corresponding to the (10), (11), and (20) reflections of a two-dimensional hexagonal lattice [27,28]. These results demonstrate that the hexagonal ordering is preserved in the PMMs, in good agreement with the microscopy observations. Complementary Fourier transform infrared (FT-IR) spectroscopy confirms that the membranes retain the chemical signatures of the polymer ligands after thermal treatment and NC removal (Fig. S2). These combined structural and spectroscopic characterizations establish that the ordered mesoporous membranes are constructed from continuous polymer frameworks, thereby providing a solid structural and chemical foundation for subsequent analysis.

**Figure 2.**
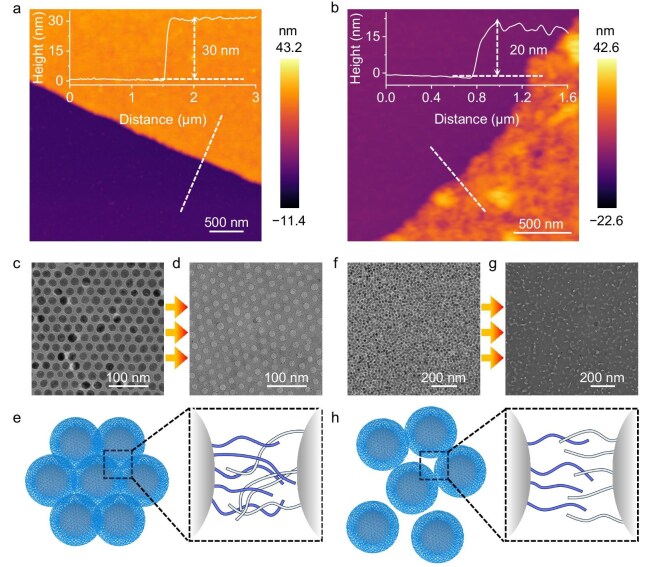
AFM topography images of (a) 2DSLs and (b) the corresponding PMMs derived from 20@13k. TEM images of (c) 2DSLs and (d) PMMs after etching. (e) Schematic illustration of physical entanglement of polymer chains in 2DSLs. TEM images of (f) disordered assemblies and (g) worm-like porous membranes formed after etching. (h) Schematic illustration of insufficient physical entanglement in disordered assemblies.

To investigate the role of confined physical entanglement between adjacent polymer-grafted NCs, AFM was used to measure the thickness of 20@13k-2DSLs and the corresponding PMMs (Fig. 2a and b). The measured thickness of 30 nm, only slightly exceeding the 20 nm NC diameter, corresponds to an effective polymer shell of ∼5 nm on each side and supports a monolayer configuration of closely packed NCs. After thermal annealing and selective etching, the PMMs show a reduced thickness of ∼20 nm. This reduction indicates that polymer layers oriented along the out-of-plane direction lack sufficient chain entanglement and are therefore removed during etching. In contrast, polymer chains confined between adjacent NCs in the in-plane direction become physically entangled during annealing, which stabilizes the lateral framework and maintains the ordered mesoporous architecture after NC removal.

To elucidate the roles of structural order and thermal treatment in the formation of mesoporous architectures, a series of control experiments were conducted using the 20@13k building blocks. When long-range ordered 2DSLs were thermally annealed and subsequently etched, well-ordered PMMs were obtained (Fig. 2c–e). In contrast, disordered assemblies prepared by rapid solvent evaporation and subjected to the same annealing and etching conditions yielded only randomly distributed, worm-like pores with low density and poor spatial uniformity (Fig. 2f–h). This difference arises from the reduced packing efficiency and lower coordination number in the disordered assemblies, which limit the extent and continuity of polymer entanglement between neighboring NCs. In addition, the ordered 2DSLs that were directly etched without prior thermal annealing yielded only isolated mesopores, although features reflecting the initial NC arrangement could still be discerned (Fig. S3). This observation indicates that long-range structural order alone is insufficient to stabilize a continuous mesoporous framework in the absence of thermally activated polymer entanglement. Together with the control experiments on disordered assemblies, these results demonstrate that confined physical entanglement induced by thermal annealing is indispensable for fixing the polymer framework during etching. Meanwhile, the 2D hexagonal packing of the 2DSLs enhances local coordination and interparticle contact, thereby facilitating the formation of robust, ordered mesoporous membranes.

Owing to the intrinsic tunability of both NCs and polymer ligands, this strategy can be readily extended across a broad parameter space of NC@PS building blocks, whose size and surface properties can be precisely controlled by adjusting the NC core diameter and the *M*_n_ of the grafted PS-PEHA ligands. By grafting PS-PEHA with *M*_n_ ranging from 2.5k to 13k onto Fe_3_O_4_ NCs with diameters of 15–20 nm, a series of well-defined NC@PS building blocks were prepared. Dynamic light scattering (DLS) measurements show narrow size distributions for the NCs both before and after ligand exchange, with polydispersity indices below 0.01 (Fig. S4). Moreover, the average hydrodynamic diameter of NC@PS increases monotonically with increasing *M*_n_ for all core sizes investigated, confirming uniform polymer grafting and enabling continuous and independent tuning of the overall dimensions of the NC@PS building blocks through ligand molecular weight. Thermogravimetric analysis (TGA) was further employed to quantitatively estimate the ligand grafting density (Fig. S5 and Table S1). For NC@PS, significant weight loss occurred above 300°C, primarily due to thermal degradation of the PS chains. Notably, the calculated grafting density decreases systematically with increasing *M*_n_ of the ligands, consistent with steric hindrance effects imposed by longer polymer chains that limit the number of ligands accommodated on the NC surface.

**Figure 3.**
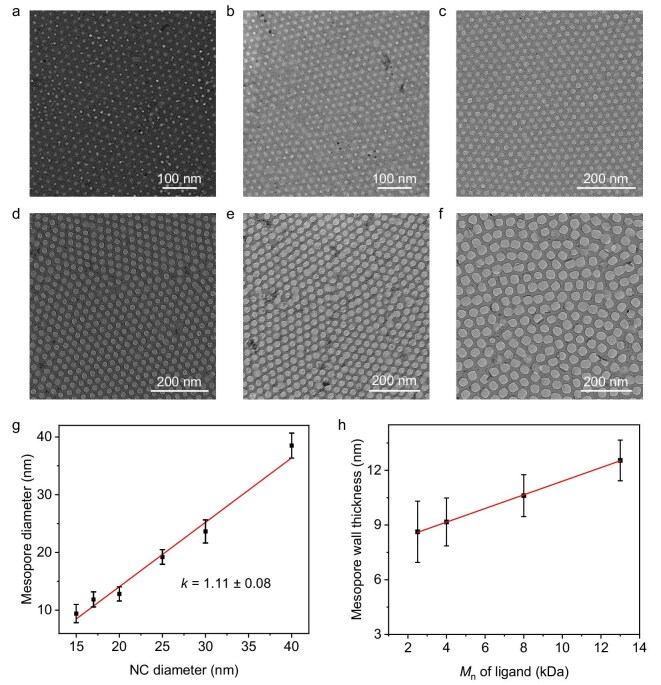
TEM images of PMMs derived from NC@PS 2DSLs with different NC sizes and polymer molecular weights: (a) 15@8k, (b) 17@8k, (c) 20@8k, (d) 25@4k, (e) 30@4k, and (f) 40@4k. (g) Correlation between pore diameter and NC size for PMMs derived from NC@4k-2DSLs. (h) Dependence of pore wall thickness on polymer molecular weight for 17@PS-PMMs.

Importantly, the formation of ordered 2DSLs and their subsequent transformation into PMMs are not restricted to a specific NC size. NC@PS building blocks with different core diameters consistently assemble into long-range ordered 2DSLs and can be converted into PMMs, yielding uniform, well-defined mesoporous structures (Figs S6–S8). To quantitatively assess the effect of NC size on the mesoporous structure, TEM was used to measure the pore sizes of the resulting PMMs. For samples grafted with the same polymer (8k PS-PEHA), a clear dependence of pore size on NC diameter is observed, with PMMs derived from 15, 17, and 20 nm Fe_3_O_4_ NCs exhibiting systematically larger pores as the NC size increases (Fig. 3a–c). Similar trends are observed for other polymer molecular weights (13k, 4k, and 2.5k), indicating that the NC size is the primary factor governing mesopore dimensions across all tested building blocks (Fig. S9). These results indicate that the NC diameter is the primary factor controlling the pore size, which can be tuned from approximately 9 to 15 nm, demonstrating that this strategy enables precise, robust control over mesopore dimensions while maintaining uniform and long-range ordered structures.

The versatility of this assembly strategy allows the incorporation of NCs with larger core sizes to construct 2DSLs and PMMs. In practice, however, Fe_3_O_4_ NCs larger than 20 nm are challenging to synthesize [29,30]. To expand the accessible size range, alternative building blocks were employed, including 25 nm NaYF_4_:Yb/Er NCs, 30 nm MnO NCs, and 40 nm NaYF_4_:Yb/Er NCs, all grafted with 4k PS-PEHA and subjected to the same interfacial assembly, thermal annealing, and etching procedures. TEM confirms that the resulting 2DSLs and PMMs maintain long-range ordered structures (Fig. 3d–f and Figs S10–S12), demonstrating the applicability of this strategy to substantially larger NCs. For larger NCs with short polymer ligands, such as 30@4k and 40@4k, partial pore wall rupture and pore interconnection are observed, likely due to the limited wall thickness and the non-spherical geometry of the NCs, which produce anisotropic interparticle contacts and heterogeneous polymer entanglement. Taken together, NC@PS building blocks with core diameters spanning 15–40 nm and grafted with 4k PS-PEHA enable precise tuning of PMM mesopore sizes across 9–40 nm (Fig. 3g). The mesopore size exhibits an approximately linear dependence on NC diameter, underscoring the key role of the NC building block in defining mesostructural dimensions. This strategy establishes a programmable route to PMMs with precisely defined and predictable pore sizes.

In addition to tuning mesopore size via NC diameter, the wall thickness of PMMs can be controlled by varying the *M*_n_ of the grafted polymer ligands. GISAXS measurements on Fe_3_O_4_ NC@PS systems with different *M*_n_ were used to quantify the interparticle spacing in 2DSLs and the corresponding mesopore wall thickness in PMMs (Figs S13–S15). For 17 nm Fe_3_O_4_ NCs, increasing *M*_n_ from 4k to 13k leads to interparticle spacings of 4.5–7.5 nm and mesopore wall thicknesses of 8.6–12.5 nm (Fig. 3h). The larger wall thickness relative to the interparticle spacing arises from relaxation of the initially constrained polymer chains upon removal of the rigid NC cores during thermal annealing and etching. For NCs of other sizes, the wall thickness generally decreases with decreasing polymer *M*_n_, reflecting the combined influence of NC curvature and polymer chain confinement, yet polymer *M*_n_ remains an effective handle for tuning wall thickness. Overall, this assembly strategy offers two orthogonal and complementary parameters for structural control of PMMs. The mesopore size is primarily dictated by NC diameter, enabling precise and predictable tuning over a wide range, while the mesopore wall thickness can be independently modulated by adjusting polymer *M*_n_. The interplay of controllable NC core dimensions and adjustable polymer shells offers a flexible and robust approach for constructing mesoporous materials with precise and tunable structural features.

**Figure 4.**
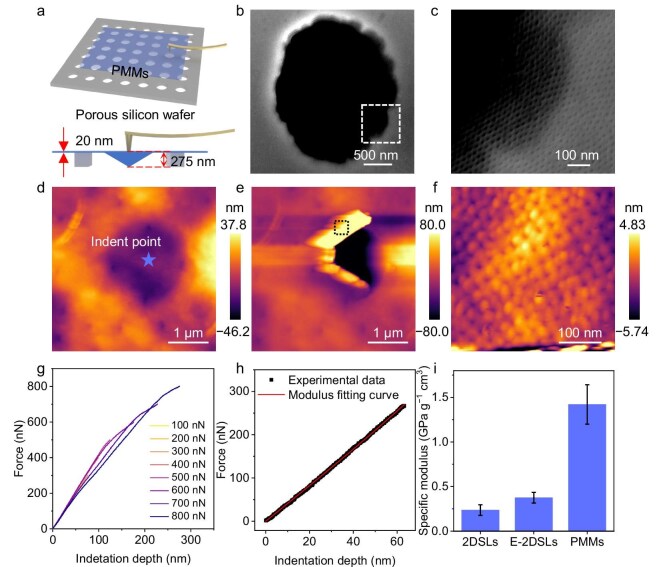
(a) Schematic of AFM nanoindentation for testing the mechanical properties of free-standing membranes. (b, c) SEM images of free-standing 20@13k-PMMs. AFM images of PMMs (d) before and (e, f) after nanoindentation. (g) Force–displacement curves recorded under different trigger forces. (h) Representative force–displacement curve used for modulus fitting. (i) Comparison of specific modulus between 20@13k 2DSLs, E-2DSLs, and PMMs.

Given the established role of physical entanglement in reinforcing polymer materials, the mechanical properties of the 2DSLs and PMMs were characterized by AFM nanoindentation (Fig. 4a), providing insight into whether such an effect is retained in the confined membrane architecture [31,32]. As a representative example, 20@13k-2DSLs were transferred from the liquid–air interface onto silicon wafers patterned with 2 μm pores, forming free-standing membranes spanning the pores. After thermal annealing to promote interparticle physical entanglement, the resulting entangled 2D superlattice membranes (E-2DSLs) were subsequently subjected to selective etching to obtain the corresponding PMMs.

Both the 2DSLs and the derived PMMs remain suspended across the pores without visible tear or collapse, indicating sufficient structural integrity for mechanical testing (Fig. 4b and c, and Fig. S16). AFM nanoindentation was performed at the center of a free-standing 20@13k-PMM membrane by applying single-point compression with gradually increasing trigger force until rupture of the membrane (Fig. 4d and e). AFM topography images acquired after fracture show that the ordered mesoporous architecture is largely preserved in the surrounding regions (Fig. 4f). Although the membranes experience localized mechanical failure at the indentation site, the nanoscale mesoporous framework remains intact. This structural resilience reflects the mechanical reinforcement provided by confined physical entanglement between adjacent polymer ligands, which helps maintain the integrity of the mesoporous architecture under indentation.

The force–displacement curves obtained from AFM nanoindentation were analyzed to evaluate the deformation behavior of the PMMs (Fig. 4g). Multiple force–displacement curves were recorded at the center of a single membrane under varying trigger forces. As the trigger force increases, the curves remain continuous, with a gradual reduction in slope reflecting yielding and progressive softening rather than abrupt breakage. This behavior indicates that the membranes accommodate increasing load through non-catastrophic deformation while largely preserving the mesoporous framework. At a trigger force of 800 nN, the PMMs deform by approximately 275 nm, corresponding to about 14 times the membrane thickness (∼20 nm), without immediate structural failure, demonstrating pronounced deformability. Similar continuous responses are observed for the precursor 2DSLs and E-2DSLs (Figs S17 and S18). Notably, the E-2DSLs maintain stable mechanical responses under high trigger forces up to 1500 nN and during repeated indentation cycles, consistent with enhanced mechanical robustness imparted by thermally activated physical entanglement.

The elastic modulus of the membranes was determined by fitting the initial linear regime of the force–displacement curves, corresponding to the elastic deformation prior to yielding. For 20@13k-PMMs, an average modulus of 1.09 ± 0.17 GPa was obtained (Fig. 4h). The precursor 2DSLs and thermally annealed E-2DSLs exhibited average moduli of 0.77 ± 0.19 GPa and 1.23 ± 0.20 GPa, respectively. The increase in modulus after thermal annealing provides direct evidence for the formation of confined physical entanglement between polymer ligands, which reinforces the polymer framework within the ordered superlattice. Following removal of the NC cores, the PMMs show a moderate decrease in modulus relative to the E-2DSLs, reflecting the reduced load-bearing cross-section due to the introduced mesoporosity while retaining the entangled polymer network.

For porous materials, absolute elastic modulus alone provides an incomplete assessment of mechanical performance, especially for low-density structures [33]. Normalizing the modulus by the effective density provides the specific modulus, which allows for a more meaningful comparison of density-normalized mechanical properties [34–36]. Using this approach, the effective densities of 2DSLs and E-2DSLs in the 20@13k system are estimated as 3.26 g cm^−3^, corresponding to specific moduli of 0.236 and 0.375 GPa g^−1^ cm^3^, respectively. In contrast, the PMMs exhibit a much lower density of 0.77 g cm^−3^ while maintaining an elastic modulus of 1.095 GPa, resulting in a substantially higher specific modulus of 1.42 GPa g^−1^ cm^3^ (Fig. 4i). These results indicate that the PMMs combine low density with robust mechanical performance, reflecting the reinforcing effect of the confined polymer entanglement. Similar trends are observed for the 20@8k and 20@4k systems (Figs S19–S21 and Table S2). For all these samples, the elastic modulus of E-2DSLs is significantly higher than that of the corresponding 2DSLs, confirming that thermal annealing induces confined physical entanglement, which stiffens the polymer framework. After removal of the NC cores, the modulus of PMMs is slightly lower or comparable to that of E-2DSLs, consistent with the introduction of mesoporosity and elimination of rigid NC cores. When normalized by density, however, the PMMs exhibit markedly enhanced specific moduli across all samples, reaching 1.6 GPa g^−1^ cm^3^, demonstrating that the combination of low density and robust polymer framework yields lightweight mesoporous membranes with superior density-normalized mechanical performance.

**Figure 5.**
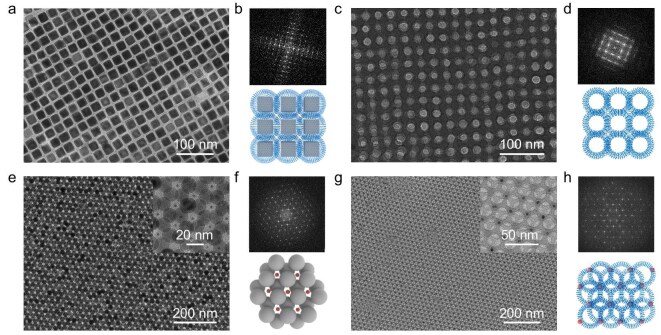
(a) TEM image of 2DSLs assembled from nanocubes@PS. (b) Corresponding FFT pattern and schematic illustration. (c) TEM image of PMMs derived from nanocubes@PS 2DSLs. (d) Corresponding FFT pattern and schematic illustration. (e) TEM images of NaCl-type binary superlattices self-assembled from 20@4k and 5@4k NCs. (f) Corresponding FFT pattern and schematic illustration. (g) TEM images of PMMs derived from the binary superlattices. (h) Corresponding FFT pattern and schematic illustration.

Taking advantage of the geometric versatility of NCs as building blocks, Fe_3_O_4_ nanocubes were employed to investigate shape-directed mesostructural control. After grafting with PS-PEHA, the nanocubes assemble at the liquid–air interface into 2DSLs exhibiting square packing symmetry, consistent with the face-to-face interaction preference dictated by the cubic geometry (Fig. 5a and b). Thermal annealing induces confined physical entanglement of the polymer ligands, thereby stabilizing the square superlattice framework. Subsequent removal of the nanocube cores yields PMMs that preserve the square lattice arrangement of mesopores, demonstrating structural inheritance from the parent superlattice (Fig. 5c and d). Although the mesopores are arranged in a square lattice, the individual pores appear approximately circular rather than square. This morphological evolution is attributed to structural relaxation of the polymer framework after core removal. In particular, polymer chains located near the edges and corners of the nanocubes experience stronger curvature-induced confinement and local stress during assembly. Upon removal of the rigid cores, relaxation of these constraints promotes reorganization of the polymer network, resulting in a rounded pore geometry.

Beyond tuning superlattice structures through NC shape control, co-assembly of NCs with distinct sizes provides an additional route to constructing more complex binary superlattice membranes [3,37,38]. Oleylamine-capped 5 nm Au NCs were prepared and functionalized with PS-PEHA (*M*_n_ = 4k), yielding polymer-grafted Au NCs denoted as 5@4k. TEM images reveal that both the pristine Au NCs and the polymer-grafted 5@4k NCs assemble into ordered superlattices (Fig. S22a and b). The significantly increased interparticle spacing after polymer grafting reflects the formation of polymer shells around the NCs, indicating that the ligand-exchange strategy remains effective even for ultrasmall NCs. DLS measurements show a clear increase in hydrodynamic diameter after ligand exchange, indicating successful polymer grafting (Fig. S22c). Ultraviolet–visible (UV–vis) spectroscopy further reveals a slight red shift of the characteristic surface plasmon resonance peak following functionalization, consistent with a change in the local dielectric environment around the Au NCs (Fig. S22d). Together, these results confirm effective polymer modification of the Au NC surface.

Binary superlattice films were fabricated by co-assembling 20@4k Fe_3_O_4_ NC@PS and 5@4k Au NC@PS at the liquid–air interface. TEM images reveal the formation of a well-defined NaCl-type binary superlattice, characterized by a highly ordered arrangement in which each Au NC is located at the center of an octahedral coordination environment formed by six surrounding Fe_3_O_4_ NCs (Fig. 5e) [5,38]. The corresponding FFT patterns confirm the long-range periodicity and structural coherence of the binary lattice (Fig. 5f). Following thermal annealing to activate confined physical entanglement of the polymer ligands, the binary superlattice architecture is effectively stabilized. Subsequent selective removal of the Fe_3_O_4_ NCs using HCl produces PMMs with ordered incorporation of Au NCs. TEM observations show that the resulting membranes consist of two mesoporous polymer layers, with Au NCs periodically arranged between them in a configuration inherited from the parent binary superlattice (Fig. 5g). In the precursor structure, the Au NCs reside within octahedral interstices defined by the surrounding Fe_3_O_4_ NCs, providing geometric confinement during annealing. This spatial constraint suppresses nanoparticle coalescence or sintering, enabling the Au NCs to remain well dispersed after core removal [39]. Owing to the high structural order of the binary template, both the mesoporous framework and the embedded Au NC array preserve long-range ordering, as further evidenced by the corresponding FFT patterns (Fig. 5h).

This strategy provides distinct structural and functional advantages compared with conventional templating and post-synthetic loading approaches. In most mesoporous polymer systems, pore size, wall thickness, and pore arrangement are inherently correlated, constraining precise and independent control over individual structural parameters. Such decoupled structural regulation remains challenging in traditional block copolymer templating or hard-template replication approaches, where pore geometry is fundamentally dictated by predefined template architectures [40]. In contrast, the present NC-based assembly decouples these parameters. Specifically, mesopore size and wall thickness are tuned by the NC diameter and polymer molecular weight, whereas pore arrangement is determined by the superlattice symmetry.

In addition to structural versatility, the resulting PMMs exhibit a relatively high specific modulus while maintaining pronounced deformability, enabling a combination of mechanical robustness and flexibility that is uncommon in porous polymer materials. This mechanical profile supports their potential use as structurally stable yet compliant porous substrates in applications such as separation membranes, flexible catalytic supports, and mechanically resilient coatings. Furthermore, the assembly strategy enables the spatially ordered incorporation of functional NCs, for example plasmonic Au NCs, directly within the mesoporous framework. Unlike conventional post-modification, impregnation, or in situ reduction approaches, which frequently result in random dispersion or nanoparticle aggregation, the superlattice-derived architecture establishes periodic and well-defined positioning of functional components throughout the membrane matrix [41,42]. The integration of mechanical resilience with long-range ordered NC organization provides a versatile platform for multifunctional mesoporous composites with potential utility in catalysis, plasmonic systems, sensing, and other advanced membrane-based technologies.

## CONCLUSION

In summary, we demonstrate an assembly-driven strategy to construct ordered PMMs through the transformation of 2DSLs enabled by confined physical entanglement. Polymer-grafted NC superlattices are stabilized by thermal annealing, which activates confined interparticle entanglement and preserves the superlattice framework during selective removal of the NC cores. Leveraging the modular nature of polymer-grafted NC building blocks, mesopore size and wall thickness can be independently tuned through the NC diameter and polymer *M*_n_, respectively. Moreover, benefiting from the rich tunability of NC size, shape, and composition as well as the structural diversity of superlattices, pore arrangement can also be independently programmed via superlattice symmetry. The resulting membranes integrate low density with robust polymer networks, achieving high specific modulus together with pronounced deformability. This work establishes a general and modular route for engineering mechanically robust mesoporous membranes with decoupled and programmable structural parameters, laying the foundation for the rational design of hierarchically organized and functionally integrated porous materials.

## METHODS

Detailed methods can be found in the supplementary information.

## Supplementary Material

nwag323_Supplemental_File
