# Supplementary material for: Confinement physical entanglement enables programmable polymeric mesoporous membranes from nanocrystal superlattices
Source: Natl Sci Rev. 2026 May 30;13(13):nwag323. doi: 10.1093/nsr/nwag323 (PMC13355320; doi:10.1093/nsr/nwag323)
Supplement: nwag323_Supplemental_File [file nwag323_supplemental_file.pdf]

Supplementary Information

Confinement physical entanglement enables programmable polymeric mesoporous membranes from nanocrystal superlattices

Zhebin Zhang[1], Ting Wang[2], Yutong Gao[2], Yifan Gao[1], Dong Yang[1], Tongtao Li[2,3,*], Wei Li[2,3,*], and Angang Dong[2,3,*]

[1]State Key Laboratory of Molecule Engineering of Polymers and Department of Macromolecular Science, Fudan University, Shanghai 200438, China.

[2]State Key Laboratory of Porous Materials for Separation and Conversion, Department of Chemistry, and Shanghai Key Laboratory of Molecular Catalysis and Innovative Materials, Fudan University, Shanghai 200438, China.

[3]Southwest Research & Design Institute of the Chemical Industry, Chengdu, China.

***Corresponding authors.** E-mails: ttli@fudan.edu.cn; weilichem@fudan.edu.cn; agdong@fudan.edu.cn

Experimental section

Materials. Styrene (>99%), ethyl α-bromoisobutyrate (EBIB, 98%), copper(II) bromide ($CuBr_2$, 99%), tin(II) 2-ethylhexanoate ($Sn(EH)_2$, 92.5-100.0%), pentaethylenehexamine (PEHA, technical grade), triethylamine (99.5%), iron(III) chloride hexahydrate ($FeCl_3·6H_2O$, 97%), oleic acid (OA, 90%), oleylamine (OAm, 70%), 1-octadecene (ODE, 90%), rare earth chlorate hydrates ($RECl_3·6H_2O$, 99.9%), ammonium fluoride (NH_4F, 98%), borane tert-butylamine complex (TBAB, 97%) were purchased from Sigma Aldrich. Sodium oleate (NaOA, 97%), tri-n-octylamine (TOA, 97%), and tris[2-(dimethylamino)ethyl]amine (Me_6TREN, 97%) were purchased from TCI. Manganese chloride ($MnCl_2·2H_2O$, 99%), tetrachloroauric(III) acid tetrahydrate ($HAuCl_4·4H_2O$, 99.9%), hexane, heptane, toluene, ethanol, tetrahydrofuran (THF), sodium hydroxide (NaOH), diethylene glycoland (DEG), and isopropanol were obtained from Sinopharm Chemical Reagent Co. Ltd (China). All chemicals were used as received unless otherwise noted. Styrene was purified by vacuum distillation prior to use. Ultrapure water (resistivity: 18.2 MΩ·cm, at 25 °C) was used in all experiments.

Synthesis of Fe_3O_4 nanocrystals (NCs). Oleic acid-capped Fe_3O_4 NCs were synthesized following a literature procedure[1,2]. First, $FeCl_3·6H_2O$ (10.8 g) and NaOA (36.5 g) were mixed with 140 mL hexane, 80 mL ethanol, and 60 mL water. The mixture was heated at 70 °C for 4 h in an oil bath to form iron oleate complexes, which were washed with water at least three times and dried under vacuum to remove residual solvents. The as-synthesized iron oleate complexes (9.0 g) and OA (1.5 mL) were then dissolved in ODE (40 mL) and TOA (20 mL) in a three-neck flask. The system was degassed under vacuum for ~2 h and subsequently heated to 330 °C under N_2 flow. After maintaining at 330 °C for 40 min, the reaction mixture was cooled to room temperature. The resulting Fe_3O_4 NCs were purified by washing with ethanol and isopropanol followed by centrifugation; this purification step was repeated twice to obtain monodisperse NCs (~20 nm in diameter). The final product was dispersed in a nonpolar solvent such as hexane or toluene. Fe_3O_4 NCs of different sizes were

obtained by adjusting the solvent ratio and reaction temperature.

Synthesis of Fe_3O_4 nanocubes. Fe_3O_4 nanocubes were synthesized following a literature procedure with minor modifications[3]. Briefly, 18 g of iron oleate complexes, 3 g of NaOA, and 5.5 g of OA were mixed with 35 mL ODE in a 250 mL Schlenk tube. The mixture was degassed at 120 °C for 1 h, then heated to 380 °C in a salt bath and maintained at this temperature for 1 h. After cooling to room temperature, the nanocubes were precipitated by adding ethanol and isopropanol, collected by centrifugation, washed, and finally redispersed in hexane to obtain a stable colloidal solution.

Synthesis of *β*-$NaYF_4$:Yb/Er NCs. *β*-$NaYF_4$:Yb/Er NCs of various sizes were synthesized following a literature procedure[4]. For the typical preparation of ~40 nm NCs, rare-earth chloride hexahydrates (total 1 mmol $RECl_3·6H_2O$; 80% Y, 18% Yb, 2% Er) were dissolved in 6 mL OA and 15 mL ODE in a 100 mL three-neck flask. The mixture was degassed under vacuum and then heated to 150 °C under nitrogen to form a clear, homogeneous solution. After cooling to room temperature, 10 mL methanol containing NaOH (2.5 mmol) and NH_4F (4 mmol) was added. The mixture was gradually heated to 110 °C to remove methanol, followed by rapid heating to 320 °C and maintained at this temperature for 1 h to promote NC growth. After cooling, ethanol was added to precipitate the NCs, which were collected by centrifugation and purified by washing with hexane and ethanol at least once to remove residual ligands and byproducts. The purified NCs were redispersed in hexane for further use. NCs with different sizes (25–40 nm) were obtained by adjusting reaction parameters such as temperature and reaction time.

Synthesis of MnO NCs. MnO NCs were synthesized in two stages—preparation of oleate precursors and subsequent thermal decomposition—following literature procedures[5]. Manganese oleate was first prepared by reacting $MnCl_2·4H_2O$ (10 mmol) with NaOA (20 mmol) in a biphasic mixture of hexane (140 mL), ethanol (80 mL), and deionized water (60 mL). The mixture was stirred and heated at 70 °C for 4 h in an oil bath. Upon completion, the organic layer containing the manganese oleate complex was separated, washed three times with deionized water, and dried under

vacuum to remove residual solvents. Monodisperse octahedral MnO NCs were then obtained via thermal decomposition of the manganese oleate precursor. Manganese oleate (4.96 g, ~8 mmol) and OA (1.12 g) were dissolved in 40 g of ODE in a 100 mL three-neck flask at room temperature. The mixture was degassed under vacuum at 120 °C for 30 min, then heated to 310 °C under nitrogen and maintained at this temperature for 1 h. After cooling to room temperature, the NCs were precipitated by adding ethanol and isopropanol, collected by centrifugation, washed, and finally redispersed in hexane to yield a stable colloidal solution.

Synthesis of Au NCs. Au NCs were synthesized following a literature procedure[6]. Typically, hexane (10 mL), OAm (10 mL), and $HAuCl_4 \cdot 4H_2O$ (0.25 mmol) were mixed and cooled to 15 °C in a thermostatic bath under a nitrogen atmosphere for 10 min. A reducing solution containing 0.25 mmol of TBAB, hexane (1 mL), and OAm (1 mL) was prepared by sonication and subsequently injected into the precursor solution. The reduction was initiated immediately, accompanied by a rapid color change to deep purple within 5 s. After stirring for 1 h, ethanol (60 mL) was added to induce precipitation of the Au NCs, which were collected by centrifugation. The purified Au NCs were finally redispersed in hexane.

Synthesis of pentaethylenehexamine-terminated polystyrene (PS-PEHA). PS-PEHA was synthesized via a two-step reaction following a literature-reported procedure with minor modifications (Scheme S1)[2,7]. In the first step, bromine-terminated polystyrene (PS-Br) was prepared using activators regenerated by electron transfer atom transfer radical polymerization (ARGET ATRP). In the second step, the terminal bromine group was converted into a multidentate nitrogen-containing ligand.

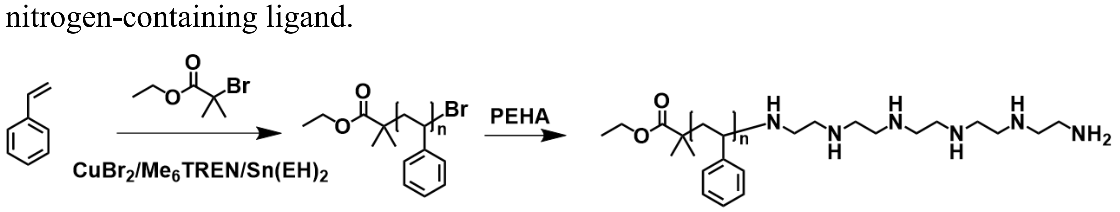

Scheme S1. Synthesis of PS-PEHA.

Typically, styrene (43.6 mmol), EBIB (436 μmol), $CuBr_2$ (4 μmol), and Me_6TREN (44 μmol) were added to a 100 mL Schlenk tube. The reaction mixture was

subjected to three freeze–pump–thaw cycles and then refilled with N_2. The sealed tube was placed in a preheated oil bath at 90 °C, and the polymerization was allowed to proceed for the desired time under stirring. After completion, the reaction mixture was cooled to room temperature, and the resulting polymer was purified by precipitation in methanol three times and dried under vacuum to afford PS-Br. To convert the terminal bromine group into a multidentate nitrogen-containing ligand, PS-Br (0.2 mmol), PEHA (10 mmol), and triethylamine (20 mmol) were dissolved in THF. The reaction mixture was stirred at room temperature for 72 h. Subsequently, PS-PEHA was precipitated in methanol and collected by vacuum filtration. This purification process was repeated several times to remove excess PEHA and small-molecule byproducts.

Ligand exchange of NCs. Typically, ethanol was added to a dispersion of 25 mg NCs in hexane to partially remove the native surface ligands. The resulting precipitate was collected by centrifugation and redispersed in toluene. Separately, 200 mg of PS-PEHA was dissolved in toluene, followed by dropwise addition of the NC dispersion under ultrasonication. The mixture was then left undisturbed for 24 h to allow efficient grafting of the polymer ligands onto the NC surfaces. The resulting PS-grafted NCs (NC@PS) were purified by precipitation with heptane, followed by centrifugation and redispersion in toluene. This purification process was repeated several times to remove excess free polystyrene and residual oleate ligands. Similar procedures were applied to prepare other NC@PS with different sizes, shapes, and compositions.

Preparation of two-dimensional superlattices (2DSLs). 2DSLs were prepared via a liquid–air interfacial assembly method, in which a dispersion of NCs was allowed to dry on the surface of an immiscible DEG liquid substrate[8]. Typically, 20 μL of a toluene dispersion of NC@PS (5 mg/mL) was dropped onto the surface of DEG confined in a Teflon well (1 × 1 × 1 cm). The well was then covered with a glass slide to slow down solvent evaporation. After 4 h, the resulting 2DSLs were transferred onto solid substrates, such as TEM grids or silicon wafers, and subsequently dried under vacuum to remove residual DEG. Binary superlattices were prepared following

the same procedure using mixed dispersions containing two types of NCs.

Preparation of polymeric mesoporous membranes (PMMs). The as-prepared 2DSLs were thermally annealed at 180 °C under a nitrogen atmosphere for 30 min to promote confined physical entanglement of the polymer ligands. Subsequently, the annealed 2DSLs supported on substrates were immersed in a 2 M HCl aqueous solution for 30 min to selectively etch the NC cores. The resulting PMMs were finally dried under ambient conditions at room temperature for further characterization.

Characterization. Transmission electron microscopy (TEM) was performed using a Hitachi HT7700 operated at 100 kV. Scanning electron microscopy (SEM) images were obtained with a Gemini SEM500 field-emission SEM at 3 kV. Atomic force microscopy (AFM) and corresponding force measurements were conducted using an Oxford Cypher VRS1250 system. Dynamic light scattering (DLS) measurements were performed at 25 °C using a Malvern Zetasizer Nano ZS. Fourier-transform infrared (FT-IR) spectra were recorded on a PerkinElmer Spectrum Two spectrometer. Thermogravimetric analysis (TGA) was conducted with a PerkinElmer Pyris 1 analyzer. Gel permeation chromatography (GPC) was performed on an Agilent 1260 Infinity II HT GPC system. Grazing-incidence small-angle X-ray scattering (GISAXS) measurements were carried out at the BL16B beamline of the Shanghai Synchrotron Radiation Facility. The X-ray wavelength was 0.124 nm. Scattering patterns were collected using a Pilatus 2M detector (Dectris, Switzerland) with a sample-to-detector distance of 2138 mm and an exposure time of 60 s.

AFM nanoindentation. AFM nanoindentation experiments were performed on free-standing membranes suspended over 2 μm diameter pores[9,10]. The porous substrates used for AFM indentation were fabricated on silicon wafers via a standard reactive ion etching process, yielding well-defined pore arrays for freestanding membrane measurements. The membrane morphology was first characterized in tapping mode. For each sample, nanoindentation measurements were carried out at the center of individual membranes. Force–displacement curves were recorded under varying trigger forces. The initial deformation regime prior to yielding was identified as the elastic region and fitted using the following equation to extract the

two-dimensional elastic modulus (E_{2D}):

$$F = \sigma_0^{2D}(\pi a)\left(\frac{\delta}{a}\right) + E_{2D}(q^3 a)\left(\frac{\delta}{a}\right)^3$$

where F is the applied force measured by the AFM system, σ_0^{2D} is the membrane pretension, a is the membrane radius, δ is the deflection at the center point, and q is a dimensionless constant related to the Poisson's ratio, typically taken as 1.02. The conventional elastic modulus of the membrane was obtained by dividing the E_{2D} by the membrane thickness.

Calculation of effective density and specific modulus. To estimate the effective density and specific modulus of 2DSLs and the corresponding PMMs, a representative hexagonal unit was selected as the repeating unit of the 2DSL, equivalent to three full NCs. The model assumes a two-dimensional geometry and neglects out-of-plane thickness variations. Using structural parameters obtained from TEM or GISAXS, including NC or pore diameter, center-to-center spacing, and pore wall thickness, the area fractions of NCs (f_{NC}) and polymer ($f_{polymer} = 1 - f_{NC}$) within the unit cell were calculated as:

$$f_{NC} = \frac{\sqrt{3}\pi D^2}{6L^2}$$

For 2DSLs, D is the NC diameter (20 nm) and L is the center-to-center spacing from GISAXS. For PMMs, D is the average mesopore diameter and L is the pore spacing from TEM. These parameters were used to calculate the area fractions of NCs or pores within the unit cell.

The effective density of the films was estimated as a weighted sum of the constituent materials based on their area fractions. In 2DSLs, both NCs and polymer contribute to the density, whereas in PMMs, the contribution of air-filled pores is negligible, and only the polymer framework is considered. The densities of Fe_3O_4 NCs and PS polymer were taken as 5.17 and 1.04 g/cm^3, respectively.

Using these density parameters together with the calculated area fractions, the effective densities of the 2DSLs and PMMs were obtained. The specific modulus was then determined by combining the effective density with the elastic modulus measured by AFM nanoindentation.

$$specific\ \ modulus = \frac{E_{measured}}{\rho_{membrane}}$$

The effective densities and specific moduli of 2DSLs, E-2DSLs, and PMMs with different polymer molecular weights were calculated using this area-fraction model, and the results are summarized in Table S2.

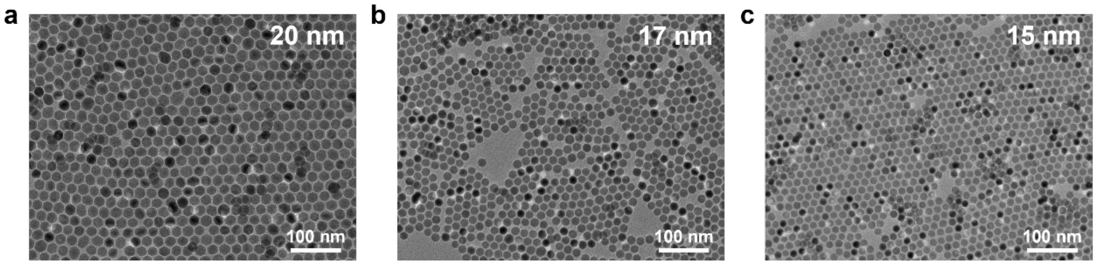

Figure S1. TEM images of Fe_3O_4 NCs with different diameter: (a) 20 nm, (b) 17 nm, and (c) 15 nm.

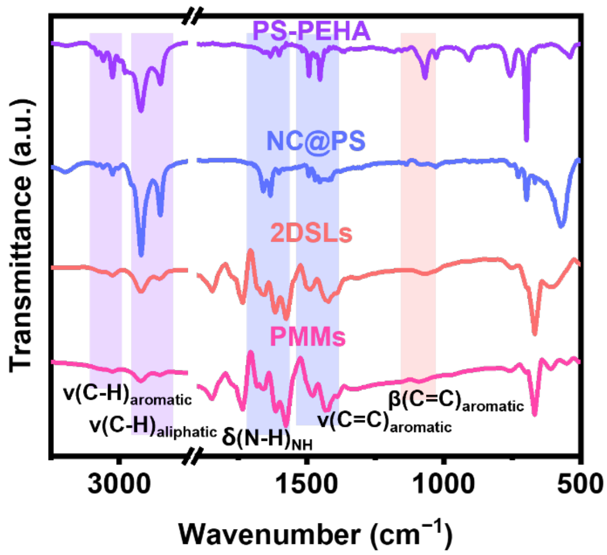

Figure S2. FT-IR spectra of PS-PEHA, NC@PS, 2DSLs, and corresponding PMMs.

FT-IR was employed to verify the ligand exchange process and evaluate the chemical integrity of the polymer framework during assembly and etching[7]. The spectrum of PS-PEHA exhibits characteristic absorption bands consistent with its chemical structure. The bands at 3100–3000 cm^{-1} are attributed to aromatic C–H stretching vibrations, while those at 2920–2850 cm^{-1} correspond to aliphatic C–H stretching. Absorptions in the range of 1700–1550 cm^{-1} are associated with vibrations of terminal amine groups, and the peaks at 1500–1450 cm^{-1} arise from aromatic ring vibrations. In addition, the band at 1069 cm^{-1} is assigned to in-plane C–H bending of the aromatic ring. The FT-IR spectra of NC@PS and the corresponding 2DSLs closely resemble that of PS-PEHA, confirming the effectiveness of the ligand exchange and the stable attachment of polymer ligands on the NC surface without noticeable desorption during interfacial assembly. Importantly, the FT-IR spectrum of the PMMs remains essentially unchanged compared to that of the 2DSLs, indicating that the etching process does not alter the chemical composition of the polymer framework.

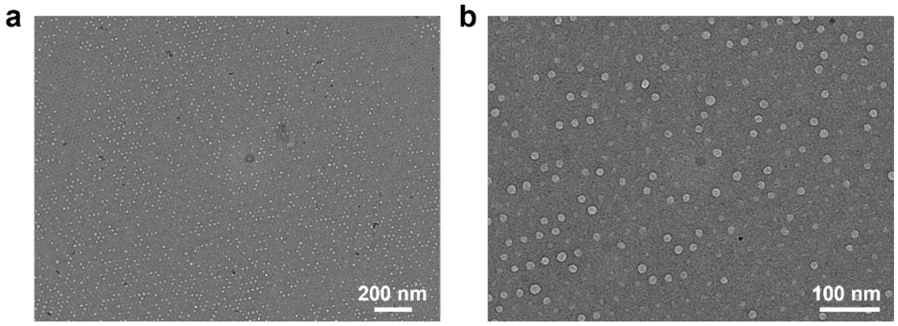

Figure S3. TEM images of membranes obtained by directly etching ordered 2DSLs without prior thermal annealing.

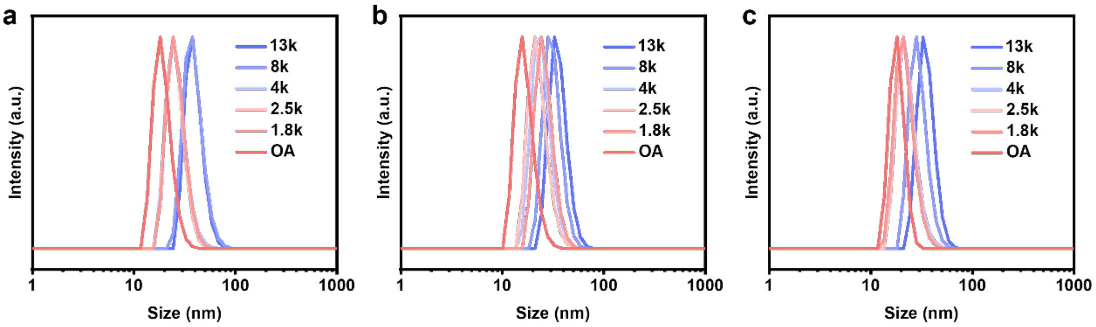

Figure S4. Hydrodynamic diameters of Fe_3O_4 NCs modified with polymers of different molecular weights: (a) 20 nm, (b) 17 nm, (c) 15 nm.

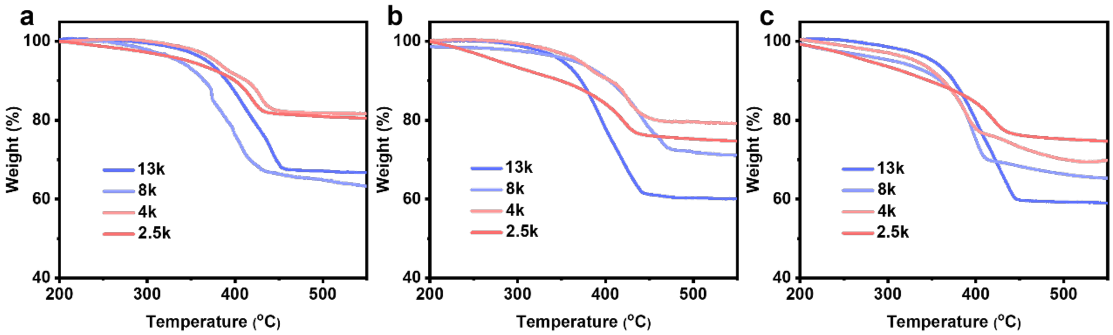

Figure S5. TGA curves of Fe_3O_4 NCs modified with polymers of different molecular weights: (a) 20 nm, (b) 17 nm, (c) 15 nm.

TGA was performed to determine the polymer mass fraction in different NC@PS samples[11]. The grafting density of the polymer ligands (σ) was then calculated using the following equation:

$$\sigma = \frac{fN_A\rho d}{6M_n(1-f)}$$

where f is the polymer mass fraction obtained from TGA, N_A is Avogadro's constant, ρ is the density of Fe_3O_4 (5.17 g·cm^{-3}), d is the NC diameter, and M_n is the number-average molecular weight of PS-PEHA. In the calculation, the NCs were assumed to be dense spheres with a density identical to that of the corresponding bulk material, and the presence of free, unbound polymer in the system was neglected. The calculated grafting density values are summarized in Table S1.

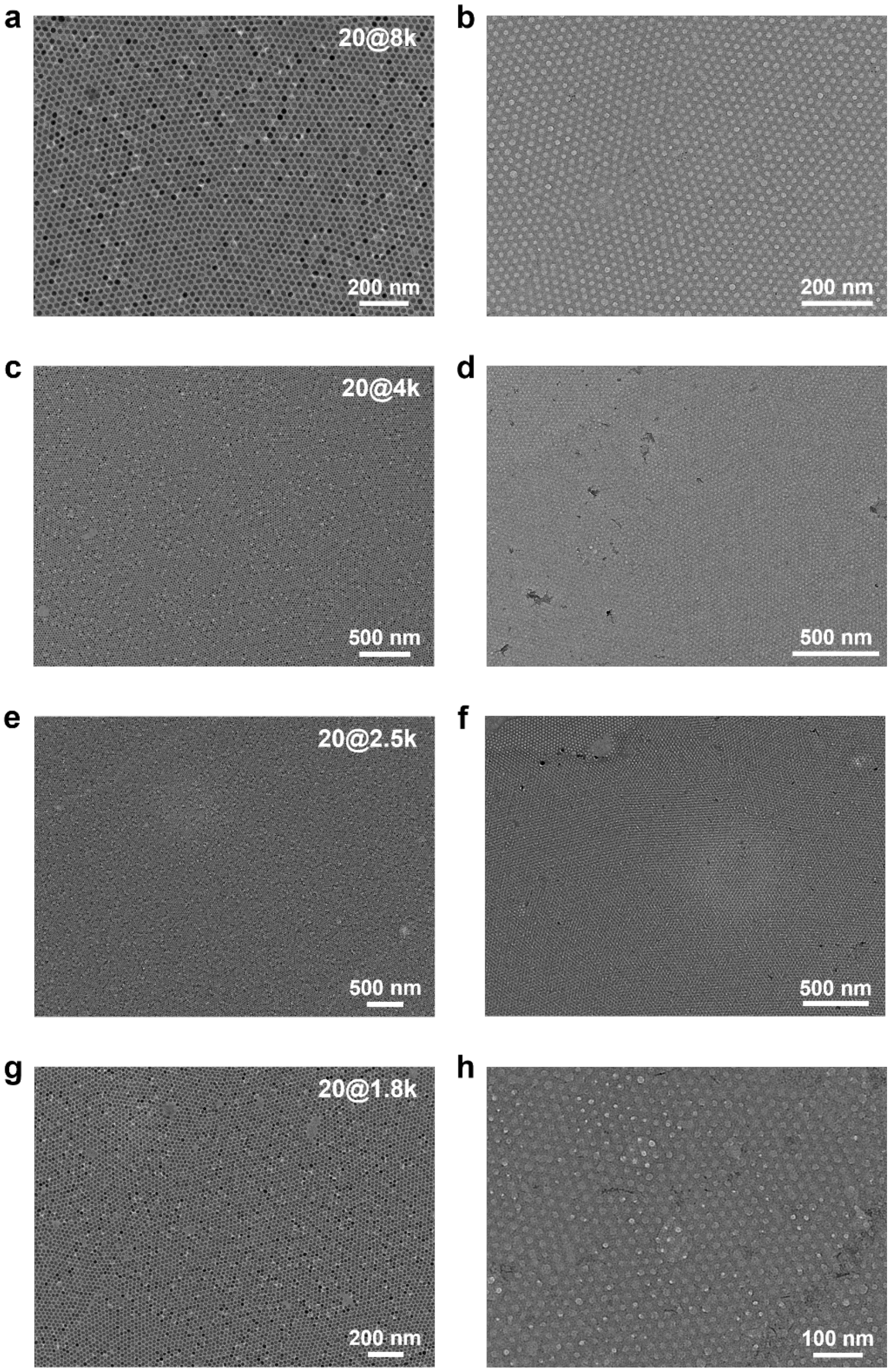

Figure S6. TEM images of 2DSLs and the corresponding PMMs prepared from different NC@PS building blocks: (a, b) 20@8k, (c, d) 20@4k, (e, f) 20@2.5k, and (g, h) 20@1.8k. In each pair, the left image shows the 2DSLs and the right image shows the corresponding PMMs.

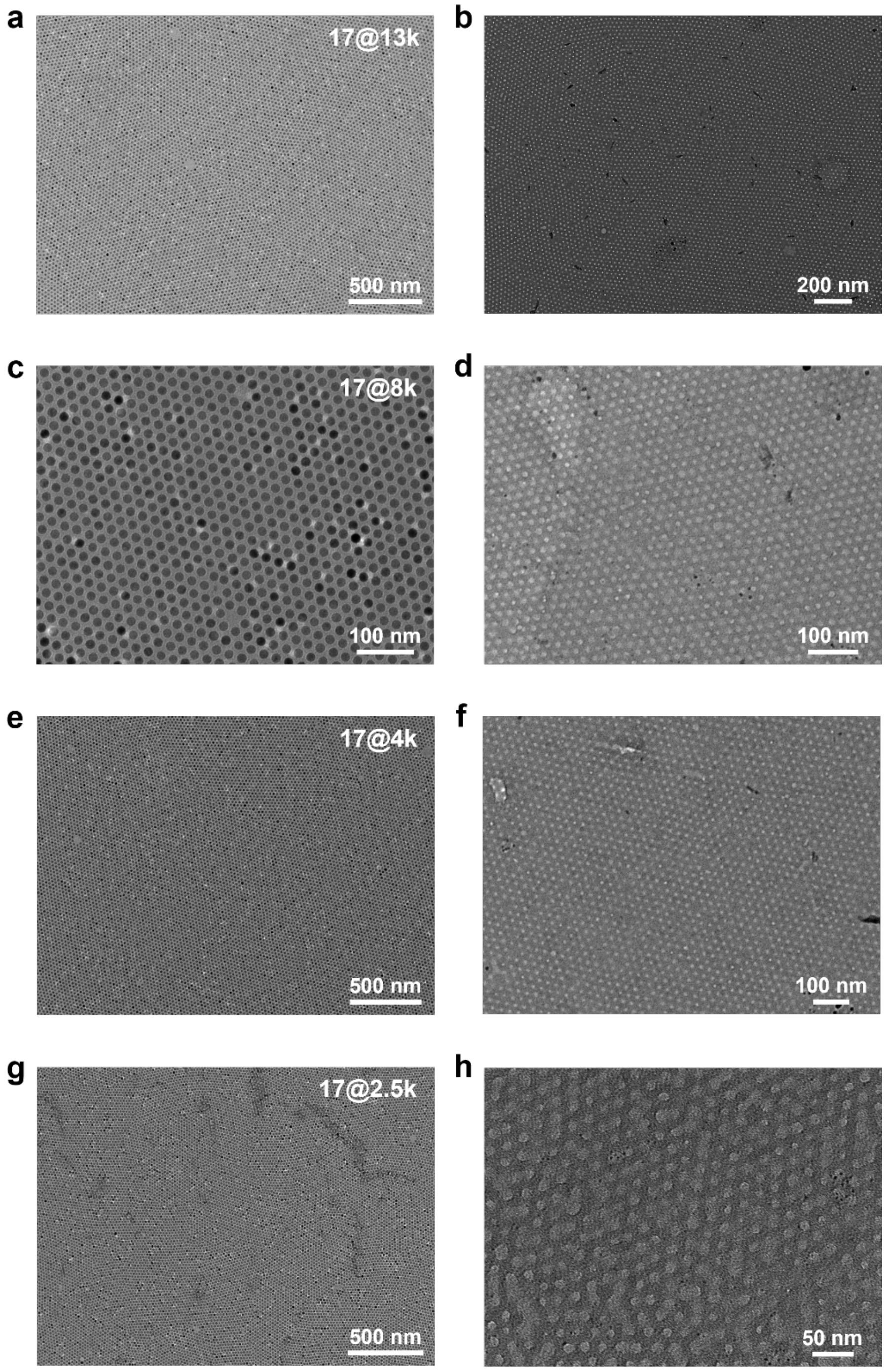

Figure S7. TEM images of 2DSLs and the corresponding PMMs prepared from different NC@PS building blocks: (a, b) 17@13k, (c, d) 17@8k, (e, f) 17@4k, and (g, h) 17@2.5k. In each pair, the left image shows the 2DSLs and the right image shows the corresponding PMMs.

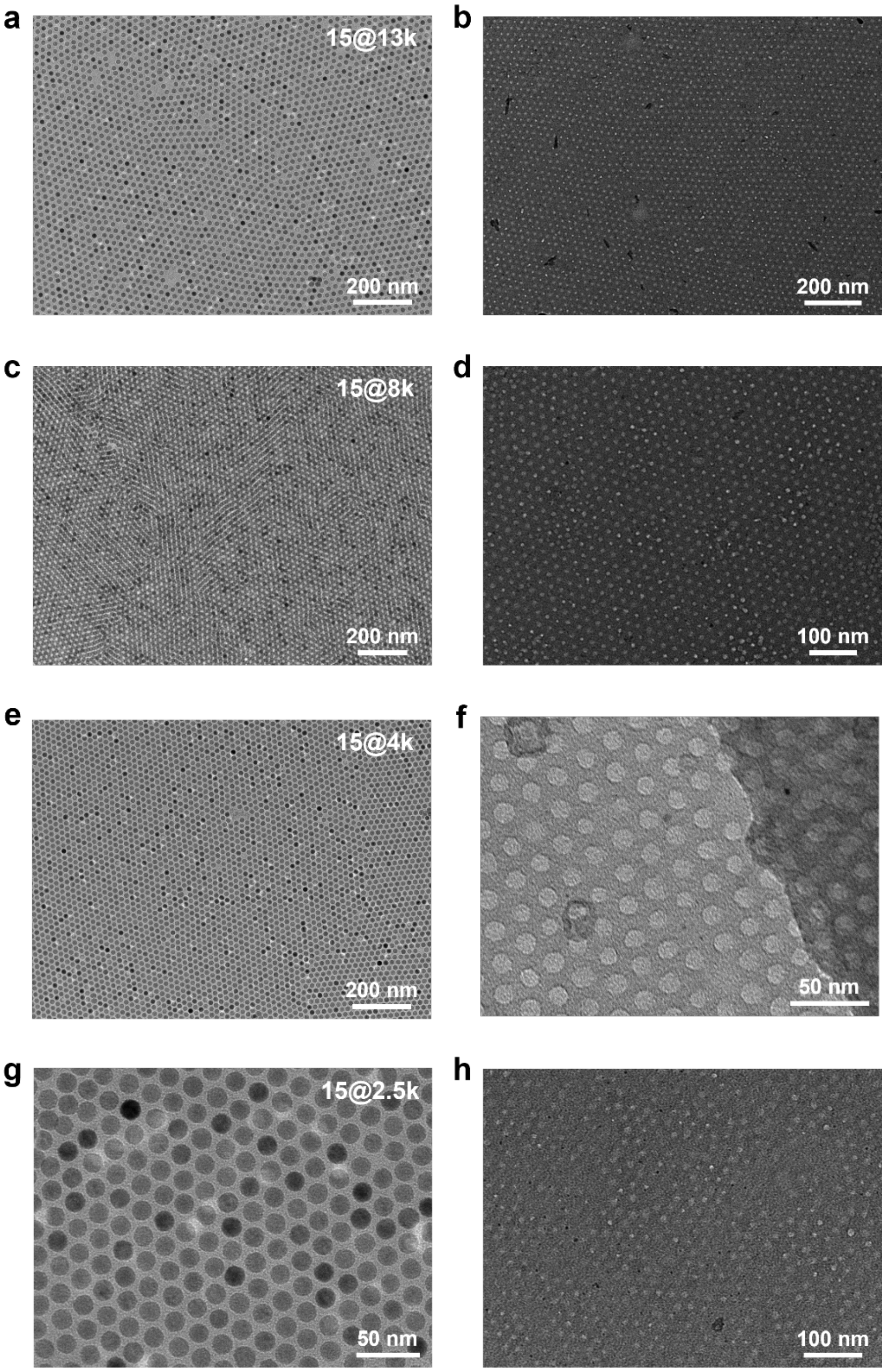

Figure S8. TEM images of 2DSLs and the corresponding PMMs prepared from different NC@PS building blocks: (a, b) 15@13k, (c, d) 15@8k, (e, f) 15@4k, and (g, h) 15@2.5k. In each pair, the left image shows the 2DSLs and the right image shows the corresponding PMMs.

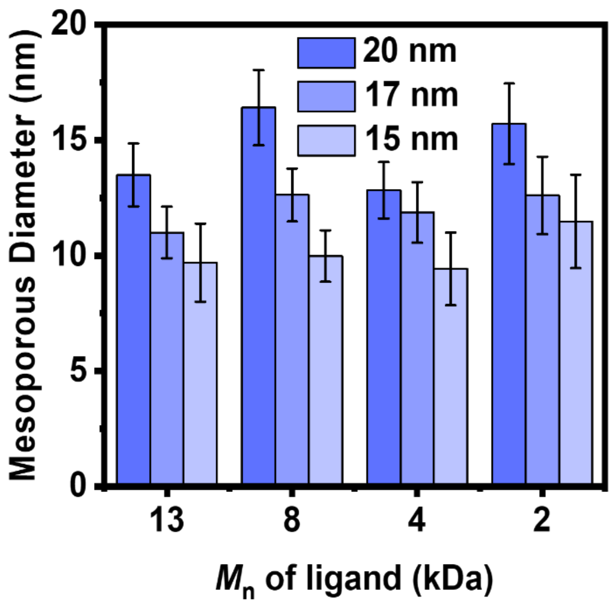

Figure S9. Statistical analysis of mesopore diameters for PMMs prepared from NC@PS with different NC diameters and polymer ligand molecular weights.

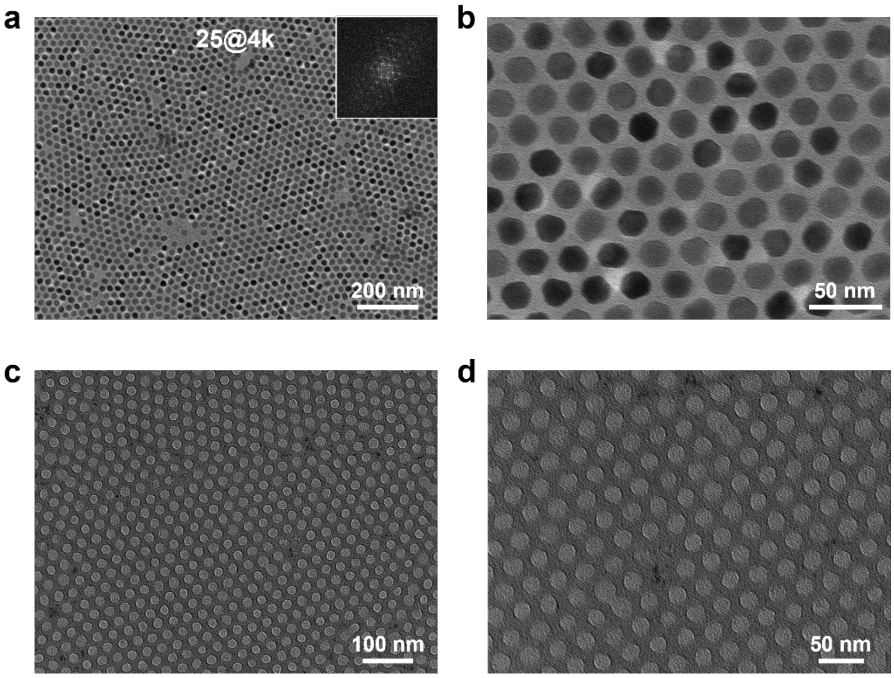

Figure S10. TEM images of (a, b) 2DSLs and corresponding (c, d) PMMs prepared from 25@4k. Inset shows the corresponding FFT pattern.

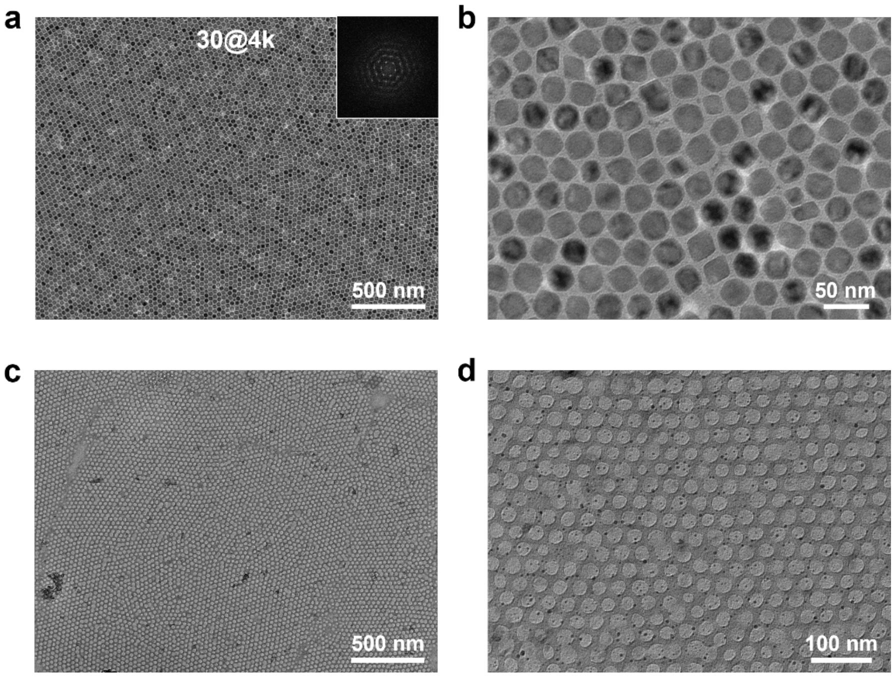

Figure S11. TEM images of (a, b) 2DSLs and corresponding (c, d) PMMs prepared from 30@4k. Inset shows the corresponding FFT pattern.

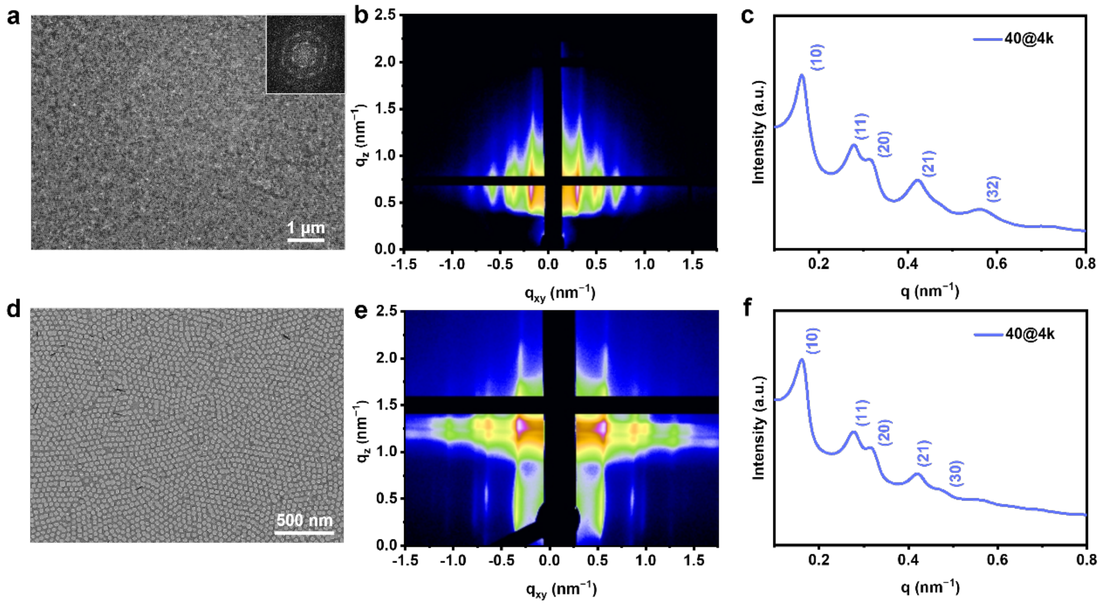

Figure S12. TEM images, GISAXS 2D scattering patterns, and 1D integrated curves of (a–c) 2DSLs and corresponding (d–f) PMMs prepared from 40@4k. Inset shows the corresponding FFT pattern.

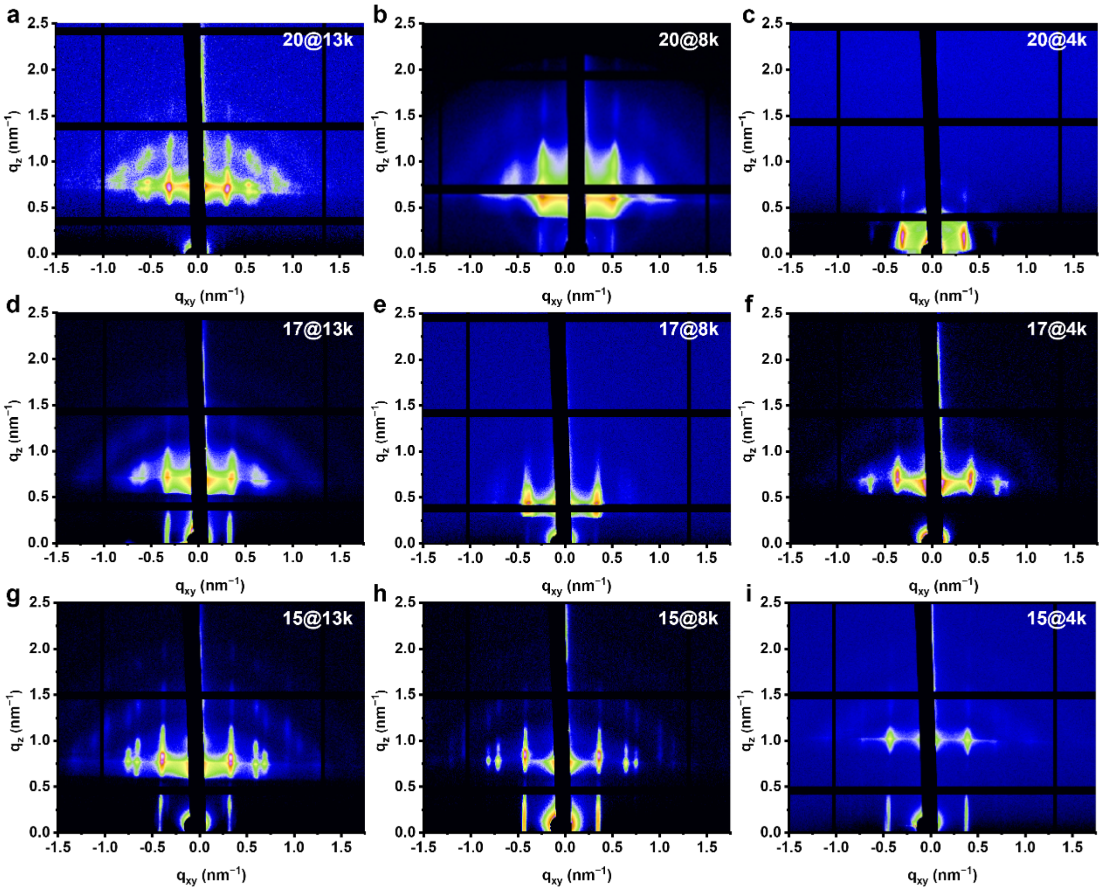

Figure S13. 2D GISAXS scattering patterns of 2DSLs prepared from (a) 20@13k, (b) 20@8k, (c) 20@4k, (d) 17@13k, (e) 17@8k, (f) 17@4k, (g) 15@13k, (h) 15@8k, and (i) 15@4k.

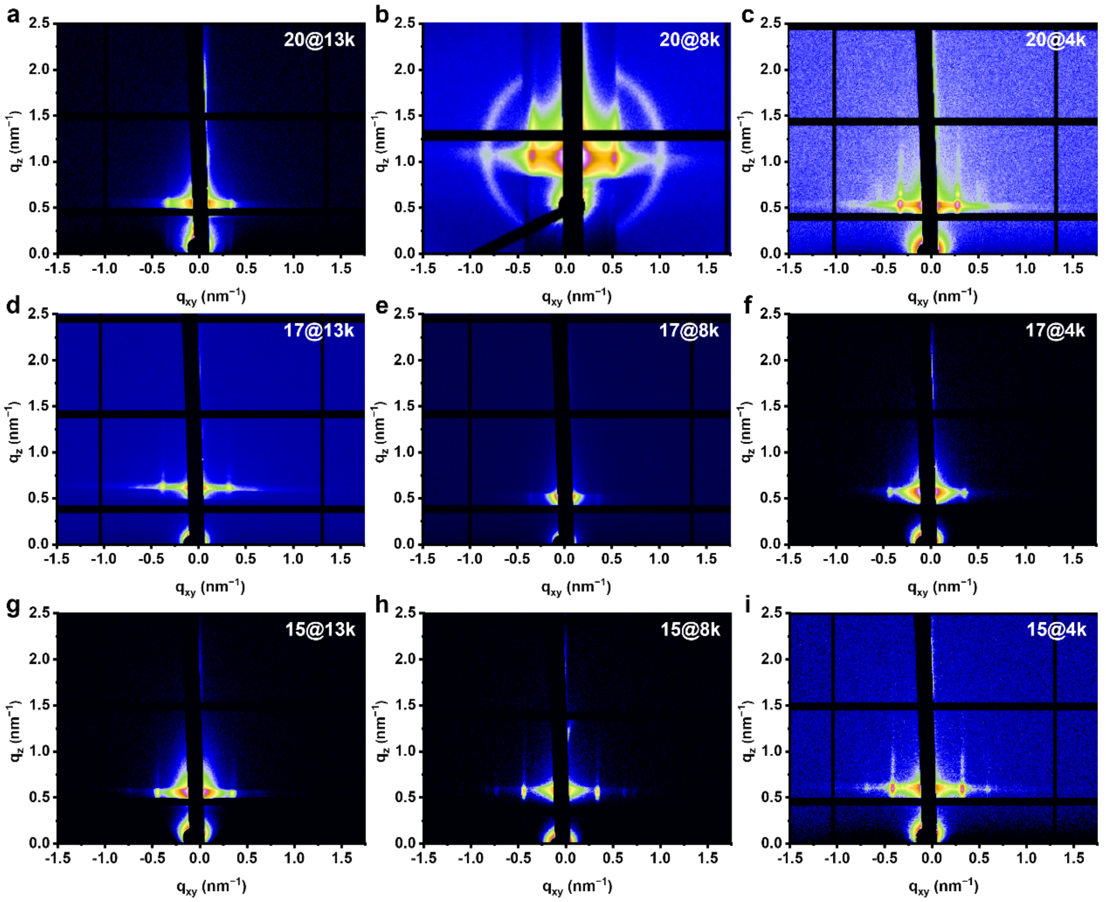

Figure S14. 2D GISAXS scattering patterns of PMMs prepared from (a) 20@13k, (b) 20@8k, (c) 20@4k, (d) 17@13k, (e) 17@8k, (f) 17@4k, (g) 15@13k, (h) 15@8k, and (i) 15@4k.

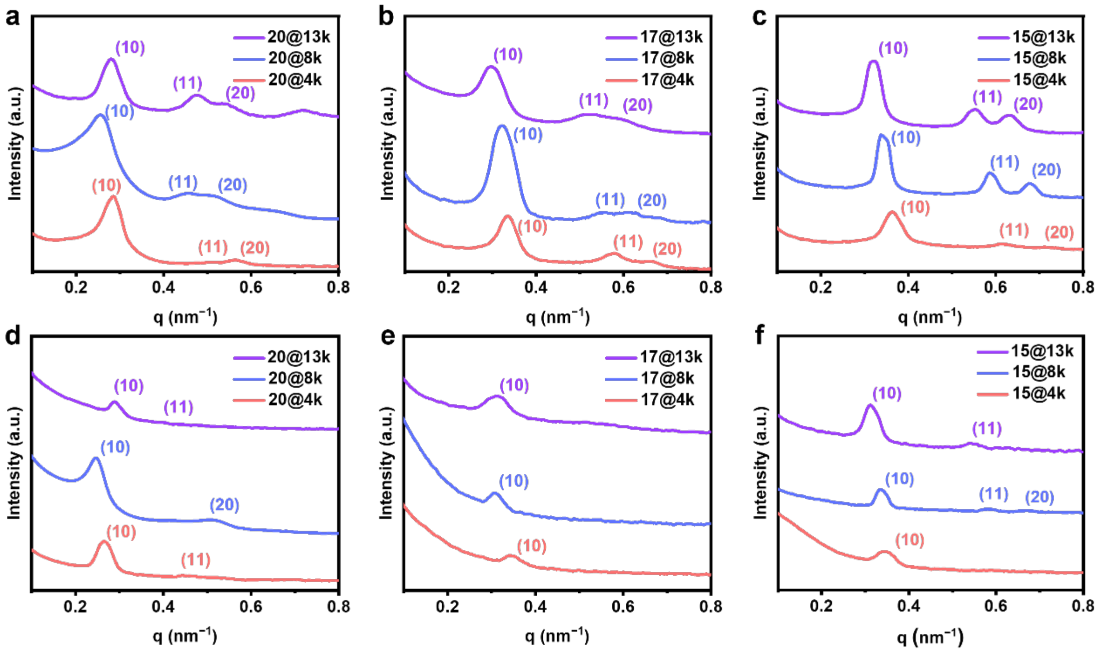

Figure S15. 1D GISAXS integrated curves of (a–c) 2DSLs and corresponding PMMs prepared from NC@PS with different NC diameters and polymer ligand molecular weights.

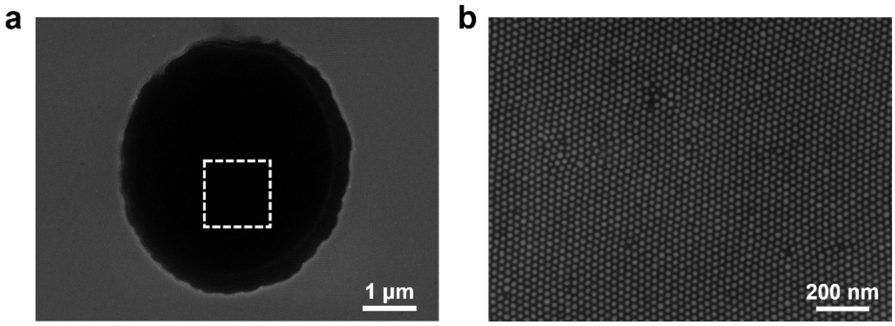

Figure S16. SEM images of free-standing 2DSLs suspended over 2 μm diameter pores.

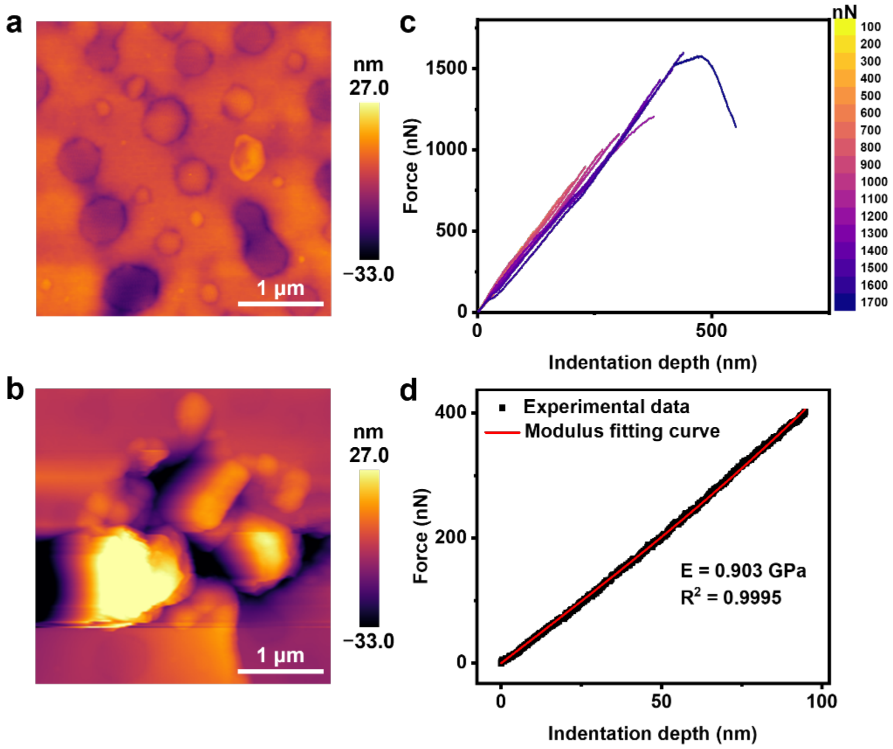

Figure S17. AFM images of 2DSLs prepared from 20@13k (a) before and (b) after nanoindentation. (c) Force–displacement curves recorded under different trigger forces. (d) Representative force–displacement curve used for modulus fitting.

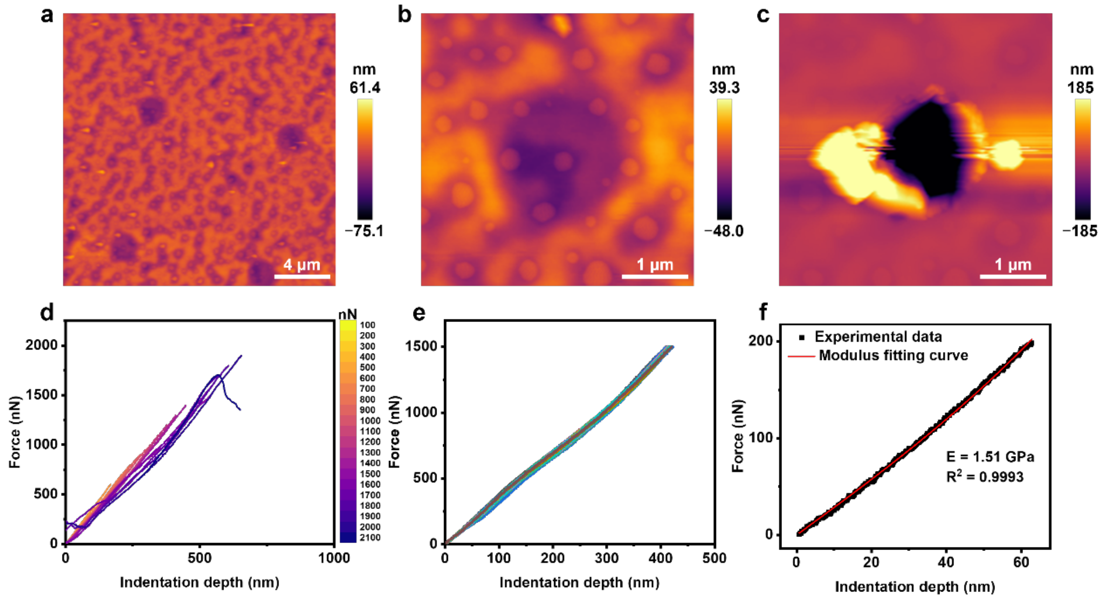

Figure S18. AFM images of E-2DSLs prepared from 20@13k (a–b) before and (c) after nanoindentation. (d) Force–displacement curves recorded under different trigger forces. (e) Force–displacement curves of 10 cycles of loading of AFM tips with 1500 nN trigger force at the same position. (f) Representative force–displacement curve used for modulus fitting.

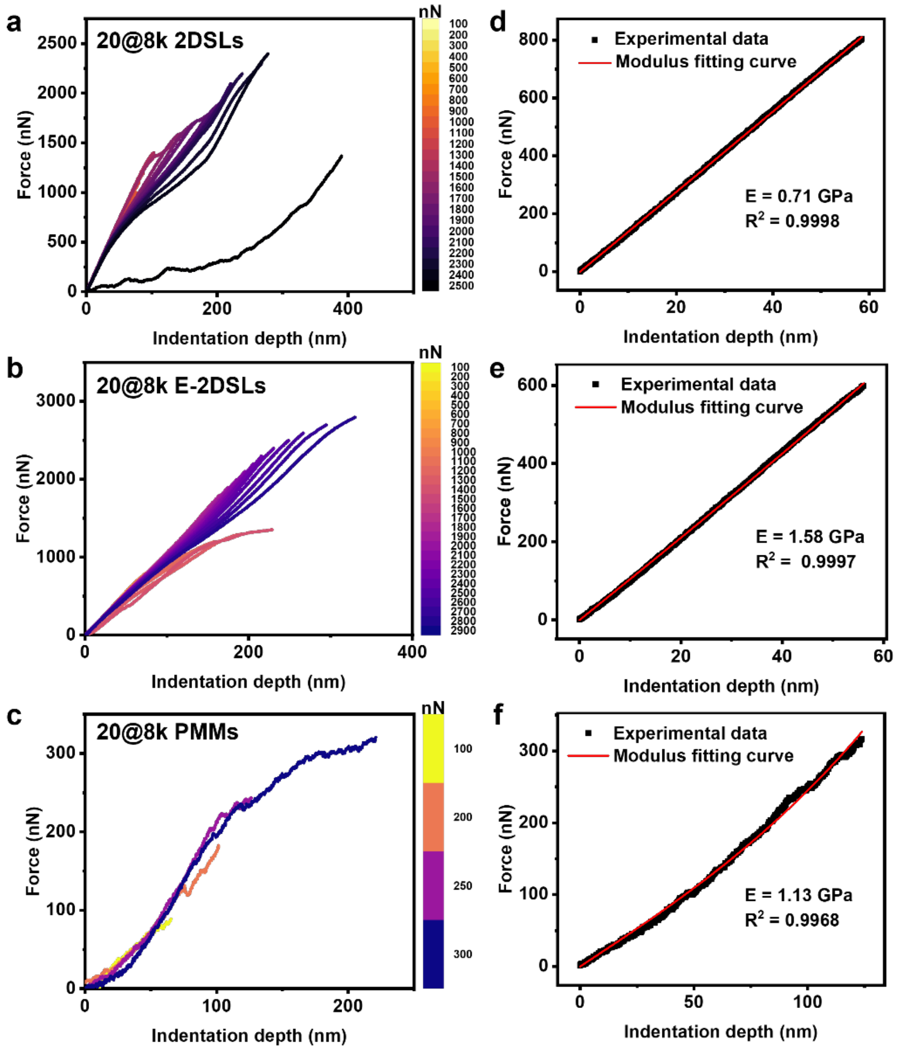

Figure S19. (a–c) Force–displacement curves of (a) 2DSLs, (b) E-2DSLs, and (c) PMMs prepared from 20@8k recorded under different trigger forces. (d–f) Representative force–displacement curves used for modulus fitting for the corresponding membranes in (a–c).

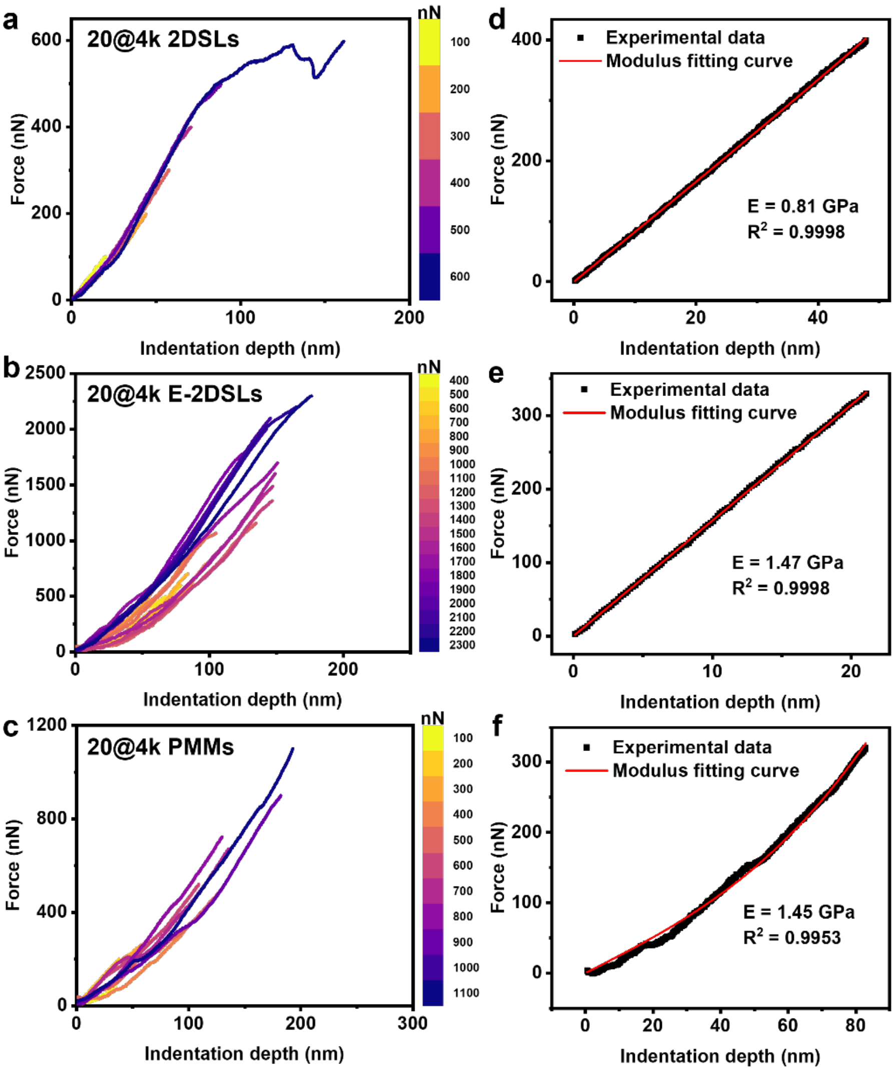

Figure S20. (a–c) Force–displacement curves of (a) 2DSLs, (b) E-2DSLs, and (c) PMMs prepared from 20@4k recorded under different trigger forces. (d–f) Representative force–displacement curves used for modulus fitting for the corresponding membranes in (a–c).

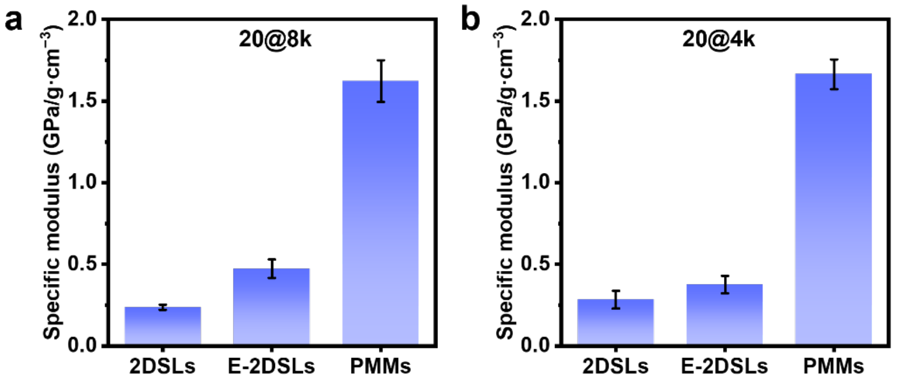

Figure S21. Comparison of the specific modulus of 2DSLs and PMMs constructed from different building blocks: (a) 20@8k and (b) 20@4k.

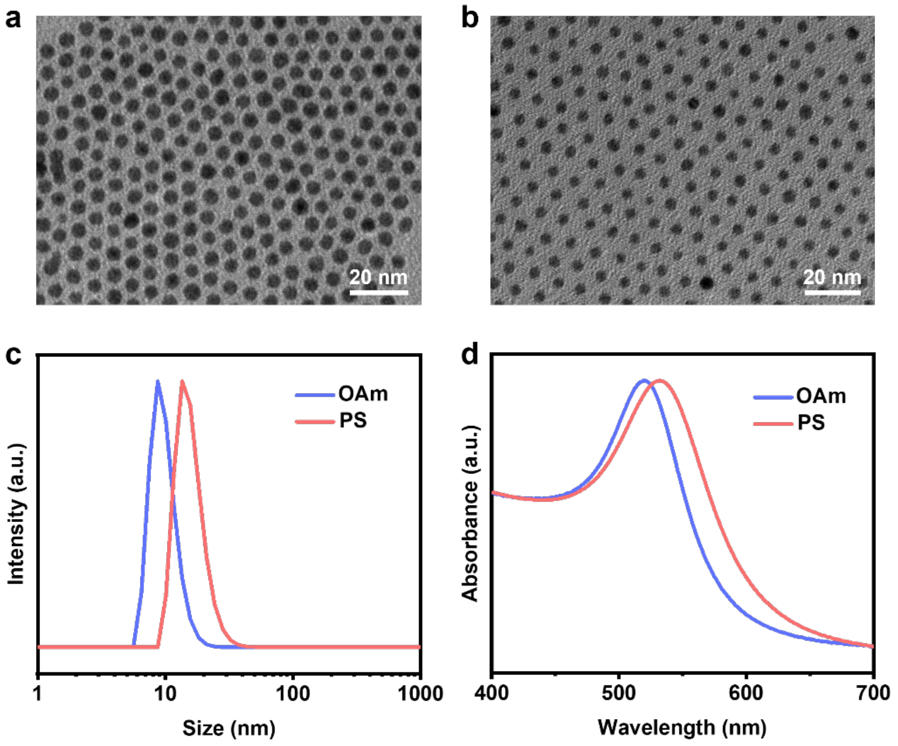

Figure S22. TEM image of 2DSLs assembled from (a) Au@OAm and (b) Au@PS. (c) Hydrodynamic diameter distributions and (d) UV–vis absorption spectra of Au@OAm and Au@PS.

Table S1. Grafting density (nm^{-2}) of Fe_3O_4 NCs modified with polymer ligands of different M_n.

M_n / NC diameter	13 kDa	8 kDa	4 kDa	2.5 kDa
20 nm	0.4	0.63	0.57	1.02
17 nm	0.44	0.41	0.57	1.49
15 nm	0.42	0.50	0.87	1.17

Table S2. Density and specific modulus of different 2DSLs, E-2DSLs, and PMMs.

Sample	$E_{measured}$ （GPa）	$\rho_{membrane}$ （$g{\cdot}cm^{-3}$）	Specific modulus （$GPa/g{\cdot}cm^{-3}$）
20@13k-2DSLs	0.7713	3.266	0.236
20@13k-E-2DSLs	1.225	3.266	0.375
20@13k-PMMs	1.095	0.771	1.421
20@8k-2DSLs	0.6875	2.911	0.236
20@8k-E-2DSLs	1.3775	2.911	0.473
20@8k-PMMs	1.2033	0.742	1.622
20@8k-2DSLs	0.9633	3.397	0.283
20@8k-E-2DSLs	1.275	3.397	0.375
20@8k-PMMs	1.38	0.829	1.664

REFERENCES

1. Park J, An K, Hwang, Y *et al*. Ultra-large-scale syntheses of monodisperse nanocrystals. *Nat Mater* 2004; **3**: 891–5.

2. Wang Y, Chen J, Zhu C *et al*. Kinetically controlled self-assembly of binary polymer-grafted nanocrystals into ordered superstructures via solvent vapor annealing. *Nano Lett* 2021; **21**: 5053–9.

3. Wang B, Wang X, Zou J *et al*. Simple-cubic carbon frameworks with atomically dispersed iron dopants toward high-efficiency oxygen reduction. *Nano Lett* 2017; **17**: 2003–9.

4. Zhang Z, Wan S, Gao Y *et al*. Monomicelle-encapsulated nanocrystals as versatile building blocks for hydrogen-bonded superlattices with enhanced mechanical properties. *J Am Chem Soc* 2025; **147**: 40051–7.

5. Li T, Xue B, Wang B *et al*. Tubular monolayer superlattices of hollow mn_3o_4 nanocrystals and their oxygen reduction activity. *J Am Chem Soc* 2017; **139**: 12133–6.

6. Wu B, Yang H, Huang H *et al*. Solvent effect on the synthesis of monodisperse amine-capped Au nanoparticles. *Chin Chem Lett* 2013; **24**: 457–62.

7. Liu Y, Klement M, Wang Y *et al*. Macromolecular ligand engineering for programmable nanoprism assembly. *J Am Chem Soc* 2021; **143**: 16163–72.

8. Dong A, Chen J, Vora PM *et al*. Binary nanocrystal superlattice membranes self-assembled at the liquid–air interface. *Nature* 2010; **466**: 474–7.

9. Zeng Y, Gordiichuk P, Ichihara T *et al*. Irreversible synthesis of an ultrastrong two-dimensional polymeric material. *Nature* 2022; **602**: 91–5.

10. Yang Y, Liang B, Kreie J *et al*. Elastic films of single-crystal two-dimensional covalent organic frameworks. *Nature* 2024; **630**: 878–83.

11. Ye X, Zhu C, Ercius P *et al*. Structural diversity in binary superlattices self-assembled from polymer-grafted nanocrystals. *Nat Commun* 2015; **6**: 10052.
